# Developing and evaluating health education learning package (HELP) to control soil-transmitted helminth infections among Orang Asli children in Malaysia

**DOI:** 10.1186/1756-3305-7-416

**Published:** 2014-09-02

**Authors:** Ahmed K Al-Delaimy, Hesham M Al-Mekhlafi, Yvonne AL Lim, Nabil A Nasr, Hany Sady, Wahib M Atroosh, Rohela Mahmud

**Affiliations:** Department of Parasitology, Faculty of Medicine, University of Malaya, 50603 Kuala Lumpur, Malaysia; Department of Community Medicine, Faculty of Medicine, University of Al-Anbar, Al-Anbar, Iraq; Department of Parasitology, Faculty of Medicine and Health Sciences, Sana’a University, Sana’a, Yemen

**Keywords:** Soil-transmitted helminth, Health education learning package, Nneglected tropical diseases, Reinfection, Orang Asli, Children, Malaysia

## Abstract

**Background:**

This study was carried out to develop a health education learning package (HELP) about soil-transmitted helminth (STH) infections, and to evaluate what impact such a package could have in terms of reducing the incidence and intensity of STH infections among Orang Asli schoolchildren in Pahang, Malaysia.

**Methods:**

To identify the key risk factors of STH in Orang Asli communities, we applied an extensive mixed methods approach which involved an intensive literature review, as well as community-based discussions with children, their parents, teachers and health personnel, whilst also placing the children under direct observation. To evaluate the package, 317 children from two schools in Lipis, Pahang were screened for STH infections, treated by a 3-day course of albendazole and then followed up over the next 6 months. The knowledge of teachers, parents and children towards STH infections were assessed at baseline and after 3 months.

**Results:**

The developed package consists of a half day workshop for teachers, a teacher’s guide book to STH infections, posters, a comic book, a music video, a puppet show, drawing activities and an aid kit. The package was well-received with effective contributions being made by teachers, children and their parents. The incidence rates of hookworm infection at different assessment points were significantly lower among children in the intervention school compared to those in the control school. Similarly, the intensity of trichuriasis, ascariasis and hookworm infections were found to be significantly lower among children in the HELP group compared to those in the control group (*P* < 0.05). Moreover, the package significantly improved the knowledge, attitude and practices (KAP) of Orang Asli people and the knowledge of teachers towards STH infections.

**Conclusion:**

A school-based health education learning package (HELP) was developed which displayed a significant impact in terms of reducing the intensity of all three main STH infections, as well as in reducing the prevalence of hookworm infections. Moreover, the knowledge levels of both teachers and the Orang Asli population regarding STH was significantly improved, a fact which greatly helped in attracting community participation and thus raising the general level of awareness regarding these forms of infections.

**Electronic supplementary material:**

The online version of this article (doi:10.1186/1756-3305-7-416) contains supplementary material, which is available to authorized users.

## Background

Soil-transmitted helminth (STH) infections, particularly those caused by *Ascaris lumbricoides, Trichuris trichiura* and hookworms, are among the most commonly neglected tropical diseases (NTDs) throughout the developing countries. Globally, about 2 billion people are infected with at least one STH species, particularly in underprivileged rural communities afflicted with poor socioeconomic status [1]. When measured in disability-adjusted life years (DALYs), STH infections are as important as malaria or tuberculosis, with 39 million life years lost to STH. Together with schistosomiasis, STH infections represent more than 40% of the morbidity caused by all tropical diseases, excluding malaria [2–4]. Numerous studies have shown that STH infections during childhood are significantly associated with protein-energy malnutrition, iron deficiency anaemia (IDA), vitamin A deficiency (VAD), poor cognitive and educational performances and poor future economic productivity [5–9].

Periodic mass deworming, proper sanitation and effective health education are three major and vital interventions for the long-term control and elimination of STH [10]. Although there are few success stories in terms of eliminating or reducing the transmission of STH in Japan, South Korea and China [11–13], the global war against worms seems to be eternal, as about 70% of schoolchildren at risk of STH infections are still not covered by deworming programmes, making the goal of complete STH eradication impossible at this time [14]. Hence, the WHO programmes and initiatives focus on the elimination of morbidity through the periodic treatment of at risk populations living in endemic areas. However, the main challenges to this strategy are that chemotherapy does not kill immature worms and thus cannot prevent the typical forms of reinfection which can occur soon after treatment [15, 16]. Moreover, there are increasing fears surrounding the possible emergence of benzimidazole drug resistance among human STH suffers, which occurs as a result of point mutations in the nematode-β-tubulin gene [17–19]. Sanitation is crucial in eliminating the overall rates of STH infections by reducing the environmental sources of such infections. However, the higher cost of proper sanitation methods compared to other interventions limits its implementation in many communities, particularly where resources are limited [20]. On the other hand, health education can be provided simply and economically, without any potential contraindications or risks, and the benefits of increased understanding of health practices among rural communities go beyond the control of helminth infections [21]. In general, providing basic information on the disease and the possible adoption of preventive measures frequently results in an increase in awareness amongst the targeted population towards specific health problem, but this does not necessarily translate to behavioural changes, which are often more difficult to achieve requiring long periods of time in order to ensure compliance with healthier habits [20].

The high prevalence of STH infections and their associated morbidities among the Orang Asli people continue to have a significant impact on public health in Malaysia. Despite the significant reduction in the overall prevalence of intestinal parasitic infections in urban areas [22, 23], the trend in rural areas, especially among the underprivileged Orang Asli populations, remains largely unchanged since the 1920s [24–31]. Moreover, the knowledge, attitude and practices (KAP) of the Orang Asli people toward intestinal helminth infections were found to be poor [32]. Within this context, developing a health education package to improve the awareness of people in regards to STH infections will help to control these infections and to save the vulnerable population from the adverse consequences associated with STH.

## Methods

### Study design

An open-label controlled intervention trial (Trial Registration: clinicaltrials.gov; identifier: NCT01640626) was carried out to evaluate the impact of the developed health education learning package (HELP) in controlling STH infections among Orang Asli schoolchildren in two primary schools in the Lipis district, Pahang, Malaysia. The schools were assigned to serve as either an intervention or control group. At baseline, a cross-sectional study was conducted with all participating children being screened for STH infections and in order to establish their eligibility in regards to taking part in the intervention study. Children from both schools were dewormed before the commencement of the intervention portion of the study was carried out. HELP was then provided to children in the intervention school only, with children from both schools being recalled for follow up examinations over the next 6 months. The time frame was set at 6 months based on our previous studies among these communities, which revealed that by 6 months of complete deworming the prevalence and intensity of STH infection were almost similar to pre-treatment levels [33, 34].

### Study area and study population

**Figure 1:**
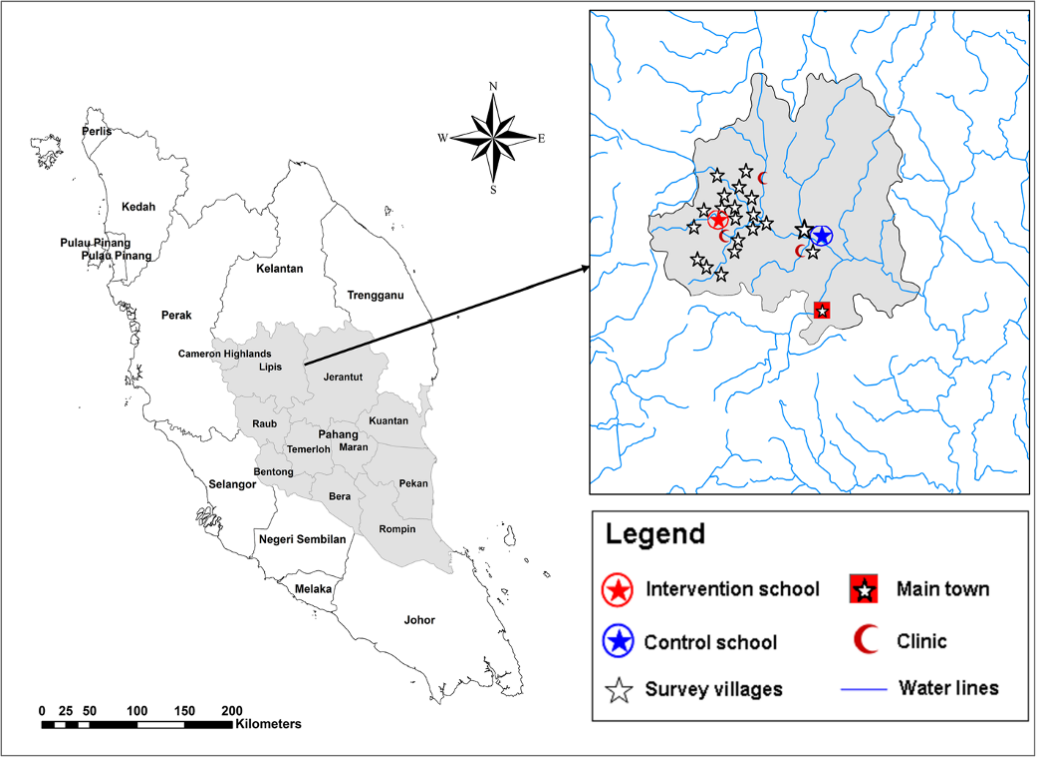
**A geographic map showing Pahang state and the locations of the selected schools and villages in Lipis district.**

This study was carried out in selected Orang Asli schools in Lipis, Pahang state, about 220 km northeast of Kuala Lumpur, with a total population of 87,200 people (Figure 1). Two primary schools in this area were selected purposively based on our previous surveys and after discussion with health officers in the Department of Orang Asli Development (JAKOA). Previous studies showed that the prevalence of intestinal helminth infections among Orang Asli children in this area was high [30, 31, 33]. Overall, two schools were selected for this study, those being Sekolah Kebangsaan Kuala Koyan (SKKK) and Sekolah Kenangsaan Betau (SKB). SKKK is located in Kuala Koyan and employs 17 teachers who look after a total of 167 schoolchildren. SKB is located in Pos Betau and employs 25 teachers who look after more than 600 pupils. The schoolchildren were from 18 villages located nearby to the schools.

A sample size of 280 children, 140 per intervention arm was estimated to give the study at least 80% power at 5% significance to detect a 10% difference in the prevalence of STH infections and intensity between the intervention group and the control group. This calculation assumed that 70% of children have intestinal parasitic infections [29–31]. An additional 20% of the calculated sample size was added to avoid the effects of dropouts and potential losses in terms of failures to attend the follow up assessments. Overall, a total of 317 children were involved in this study (172 from SKB and 145 from SKKK).

### Data collection and empirical methods

The development of HELP was carried out from January to December 2012. The baseline data collection included screening eligibility and testing for STH infection, questionnaires to reveal KAP regarding STH. The collection of data surrounding demographic, socioeconomic and environmental factors was conducted between January and March 2013, while the intervention study for evaluating the package was conducted between April and October 2013 with a monthly assessment and follow-up.

### Development of HELP on STH infections

At the beginning, we drew on several conceptual models to ensure that our efforts covered all possible situations. Thus, we relied on the PRECEDE-PROCEED model that was developed and introduced in 1974 by Dr. Lawrence W. Green to help health programme planners, policy makers and other evaluators analyze situations and design health programmes efficiently [35]. The model suggests starting the process with diagnostic planning to evaluate cultural, behavioural, environmental and educational factors that may influence the efforts to control STH infections. Hence, group discussions were held with experts from the field of Parasitology, Public Health, Education, Psychology and School Health, as well as the Department of Orang Asli Development, education office, and other researchers. A household survey that included household observations, infrastructure assessments and group discussions with schoolchildren, parents, teachers and health clinic personnel was also conducted. In addition, group discussions were held with animation design experts and animation production companies in Kuala Lumpur, Malaysia. We also considered the importance of the target population’s beliefs and attitudes about diseases, specifically how these beliefs may influence perceived capabilities and approaches towards preventing STH infections, and whether or not providing health education would actually be an effective means of reducing their risk of such infections. These factors were crucial in order to develop a package that met the needs of the target population, as they allowed the research team to properly assess ways to actively encourage these people to participate effectively in the overall efforts.

The development of HELP was conducted in three phases as follows:

### Identification of the key risk factors of STH infection among Orang Asli children

This was done through an intensive review of the previous epidemiological studies conducted among Orang Asli communities. Moreover, we visited many Orang Asli communities in different states which enabled us to report the presence of some risk factors through direct observations and by group discussions with Orang Asli residents. Pooled risk factors identified by the previous studies and confirmed by our observations were then analyzed to identify the key risk factors.

Several previous studies were conducted in rural Malaysia which showed that poverty (low household monthly income), being below 10 years of age, lack of toilet facilities, a lack of safe drinking water in a household, low educational levels of parents, poor personal hygiene (including not washing hands before eating and/or after defecation), not washing fruits and vegetables before consumption, and walking barefooted when outside the family home were all significant risk factors for contracting STH infections among Orang Asli people [29–31, 34, 36]. During our visits to the villages and schools, we observed that about half of the houses lacked functioning toilets and/or a lack of clean tap water. We also observed that most Orang Asli children practice open defecation and preferred to defecate in the local rivers, including those who have access to fully functioning toilets in their homes.

We noted a high number of flies in these communities. Flies have also been identified as mechanical vectors of intestinal parasitic infections [37]. Moreover, Sulaiman *et al.* [38] reported that the dominant fly species *Chrysomya megacephala*, collected from rural areas in Malaysia, carried eggs of *A. lumbricoides*, *T.trichiura* and hookworm, as well as the filariform infective larvae of hookworm on their external body surfaces and in the gut lumen.

### List of health messages to be delivered to the target population

The key health messages were formulated according to the identified key risk factors. The messages were simple in order to ensure they would be properly understood by the target population. We found that most of the risk factors were related to personal hygiene, while only some were related to demographic and socioeconomic factors. Hence, HELP focused more on personal hygiene practices, with demographic factors (such as being aged < 10 years) were only used for recommending intensification of control measures among certain age groups. Overall, the key messages for prevention created for the present study were:

1. Washing hands before eating.
2. Washing hands with soap after playing with soil.
3. Washing hands with soap after using the toilet.
4. Wearing slippers or shoes when going outside.
5. Avoiding open (indiscriminate) defecation.
6. Washing vegetables and fruits before consumption.
7. Drinking clean (boiled) water.
8. Covering food from flies.
9. Cutting nails periodically.

### Identification of means to deliver the messages to the target population

The key health messages were integrated into a health education learning package which involved a workshop for teachers, teacher’s guide book on STH, posters, a comic book, drawing activities, a sanitary bag, puppet show, 2 nursery song videos and group discussions. At the beginning, we decided on a mascot to be displayed across all publications in order to reinforce and reiterate the important health messages to pupils. The comic book, posters, sanitary bag and music videos were designed and produced by Animagis Sdn Bhd, Malaysia.

In the present study, the concepts related to STH and HELP were provided to the teachers from the intervention school (SKB) in the form of a half-day workshop which involved an assessment of the teachers’ knowledge about STH, followed by a session in which they had the opportunity to observe microscopic slides of STH ova and larvae, a worm gross specimen session, a scientific posters show session, capped off with a scientific lecture on the STH infections. Moreover, a teacher’s guide to STH booklet was distributed to the teachers, with further training being provided to help them understand how to assist in the introduction and follow-ups of the package. With regards to the posters, three posters were produced to convey the main health messages relating to STH and were fixed on walls all over the school (Additional file 1). Besides that, a comic book consists of 18 coloured pages, designed and printed to appeal to Orang Asli children in a brief, simple and attractive way so as to help them recognize the risk factors, consequences and preventive measures in regards to STH infections (Additional file 2). The lyrics of two nursery songs (the first song was about how to wash your hands properly, while the second resembled the comic book’s characters and story) were provided on the internal covers of the comic book. The songs were installed on computers in the school computer lab (30 desktops). The songs are available online via YouTube [http://youtu.be/aBy2TPEQquM and http://youtu.be/UNd3Q89EaH8]. In the present study, a puppet show was prepared at the Department of Parasitology, University of Malaya using good quality set pieces and well crafted hand puppets. Moreover, drawing activity on the intestinal worms was carried out at both schools; at baseline and again after 3 months. In order to help children to practice what they have learned and as a reflection on the abbreviation of this package (“HELP”), each child was given a sanitary bag which contained slippers, hand soap and nails clipper together with the comic book and the posters. The nine health messages were also displayed on both sides of the bag to help reinforce and reintroduce the messages to children through multiple means.

### Evaluation of HELP to control STH infections

#### Questionnaire survey

The parents of children were interviewed face-to-face in their homes so as to fill out a pre-tested questionnaire adopted from a previous study [31]. The questionnaire was designed in two parts. The first part centred around demographic, socioeconomic and environmental data, as well as personal hygiene, habits and health status. The second part revolved around the parents’ KAP in regards to STH. Questions in the knowledge section were designed to test the understanding of respondents on the subject of STH. These were open-ended questions, without multiple-choice answers, as such options can result in guessing and therefore give a false impression as to the knowledge of the population. Questions on attitude were designed to investigate the prevailing attitudes, beliefs and misconceptions of the population about STH. Questions in the practice section were designed to assess the practices of the population with regards to STH.

Two research assistants from JAKOA and from the Department of Parasitology at the University of Malaya were trained on how to administer the questionnaire for the purpose of this study*.* During the interviews, observations were made on the personal hygiene of the children and household cleanliness in general, including the availability of functioning toilets, piped water, cut nails, the use of footwear when outside the house, as well as general hand and clothing cleanliness.

### Stool examination

Fresh faecal samples were collected from each participant at baseline, again at 12–14 days after treatment and again monthly over the next 6 months. The faecal samples were collected into 100 ml clean containers with wide mouths and screw-fit caps before being transported (within 5 hours of collection) in suitable cool boxes at temperatures between 4 and 6°C for examination at the stool processing laboratory in the Department of Parasitology, Faculty of Medicine, University of Malaya. The samples were examined by Kato-Katz technique for the presence of STH eggs and by Harada Mori culture technique to detect hookworm larvae in light infections [2, 39]. Egg counts, as a measure of worm burden, were also carried out using the Kato-Katz technique and the results were recorded as eggs per gram (epg) of faeces. Results were graded as heavy, moderate or light according to criteria proposed by the World Health Organization [2]. For *Ascaris*, *Trichuris* and hookworm infections, egg counts of ≥ 50,000 epg, ≥ 10,000 epg and ≥ 4,000 epg, respectively, are regarded as heavy infections while egg counts of < 5,000 epg, > 1,000 epg and > 2,000 epg, respectively, are regarded as light infections.

### Deworming treatment

After baseline screening for the presence of intestinal parasitic infections, infected children were listed accordingly and received a 3-day course of 400 mg/daily albendazole tablets. Albendazole tablets (GlaxoSmithKline, London, UK) were ordered from the manufacturer’s representatives in Malaysia. The available tablets were of 200 mg each, enclosed in a sachet of two tablets. Each child was given 2 tablets at a time to chew with a chocolate biscuit while being observed by a researcher, medical officer and a teacher (Direct Observed Therapy). There were no complaints from the children during the period of being given albendazole.

### Follow up

**Figure 2:**
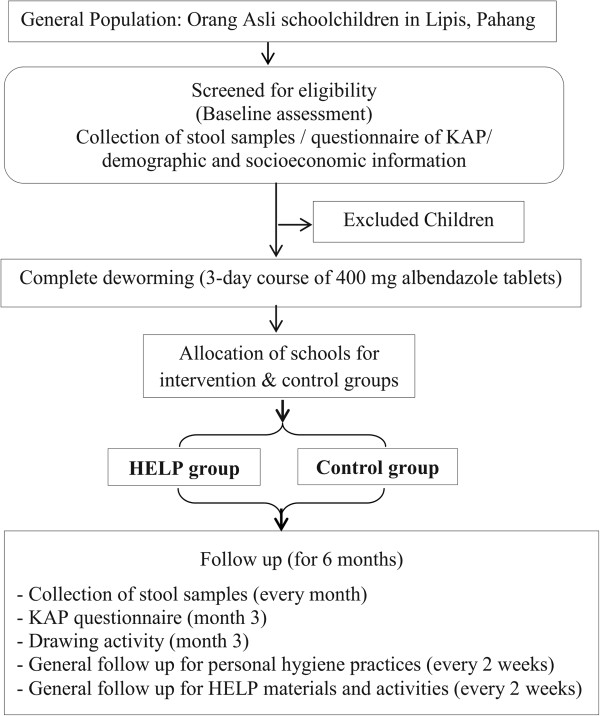
**Flow chart of the study’s activities and follow up.**

The follow up of HELP activities were performed regularly by visiting the school and villages every two weeks, during which time students were reminded of what they had been taught about STH and were encouraged to practice what they had learned. For nail clipping, a weekly follow up by the teachers was performed and the results were recorded for our researchers. The story concepts and scenario presented in the comic book were discussed twice a week by the teachers, once in the class room and once in the library. With regards to the songs, students recited and sang both songs once a week in the computer laboratory. In addition, all the children were instructed to fix the posters around their homes and to extend the information about STH to their families, siblings and friends from their villages. For instance, children were asked to educate their mothers on the importance of washing vegetables and fruits before consumption, and to keep reminding family members to wash their hands before eating. For the slippers, children were instructed to wear the slippers given to them at their villages, especially when walking or playing outdoors. Throughout many visits to the villages, the research members followed up on these issues. HELP materials were checked and replaced when needed. Overall, we found that HELP was well-received, with effective contributions and high levels of interest from teachers, children and their communities. The steps involved in the evaluation of HELP are shown in Figure 2.

### Statistical analysis

Data analysis was performed using SPSS (version 13). Data was reviewed and double checked before and after data entry. For descriptive analysis, prevalence of infections and illnesses are expressed in percentages, while mean (standard deviation; SD) or median (interquartile range; IQR) values are used to present the quantitative data, with the results being presented in tables and figures. All quantitative variables were examined for normality by Kolmogorov-Smirnov Z test before analysis. Egg counts were found to be not normally distributed, however, there are biological justifications for using the arithmetic mean rather than the median or geometric mean to express the egg counts of each STH species [40, 41]. The analysis of STH reinfection was based on children who were either initially uninfected or who had no parasite eggs in their faecal samples after complete deworming [33, 42].

For inferential statistics, the dependent variables were the prevalence and intensity of infections, while the independent variables were demographic factors (age, gender and family size), socioeconomic factors (parents’ educational levels and employment status and household monthly income, having a toilet in the house, etc.), and personal hygiene practices (washing hands before eating, washing fruits & vegetables before consuming, indiscriminate defecation, etc.). Each component of the KAP questionnaire were compared according to the STH infection status and the independent variables, as well as by using Chi-square test or Fisher’s exact test where applicable. Odd ratios (ORs) and 95% confidence intervals (CIs) were computed. In order to control the variation of the number of children in each household, weight cases, derived by the sampling fraction 1/number of participating children from each family, was used in order to analyse the data for the impact of HELP on the KAP.

To investigate the impact of the health education package on STH infections, the prevalence of STH infections were compared between the intervention group (SKB) and the control group (SKKK) by using a Chi-square test and an intention-to-treat approach for data analysis. Similarly, a Mann Whitney U test and Wilcoxon rank-sum test were used to compare the intensity of infections (mean epg) between the groups.

### Ethical consideration

The present study was carried out according to the guidelines laid down in the Declaration of Helsinki and all procedures involving human subjects were approved by the Medical Ethics Committee of the University of Malaya Medical Centre, Malaysia (Reference Number: 932.7). Written and signed (or thumb-printed) informed consent was obtained to conduct the study from parents/guardians on behalf of their children before starting the survey. All the infected children were treated with a 3-day course of 400 mg albendazole tablets.

## Results

### General characteristics

Faecal samples were collected from 317 schoolchildren (48.9% males and 51.1% females) aged between 6 and 12 years, with a median age of 9 years (IQR = 8, 11). Overall, 172 and 145 children from SKB and SKKK respectively were involved in this study. Poverty is predominant in these communities, with about two thirds of the families having a low monthly income (<RM500) that equated to being below the poverty income threshold for Malaysia. Moreover, 42.3% and 56.2% of the fathers and mothers, respectively, had no formal education. Only 30.6% and 5.7% of the fathers and mothers, respectively, were working; mainly as farmers or workers in rubber and oil palm plantations, forestry, fishing and other related occupations. Almost half of the houses (47.9%) were without toilets and 46.7% were without a piped water supply.

### Intestinal parasitic infections at baseline

Overall, 99.4% of the children were found to be infected by at least one parasite species. Of those infected, 61.0% had mixed STH infections while 39.0% had single infections. The prevalence of trichuriasis, ascariasis and hookworm infections were 96.2%, 51.4% and 34.1% respectively. Almost two-thirds of the trichuriasis and half of the ascariasis infections were of moderate-to-heavy intensities, while all hookworm infections were of light intensity.

### Evaluating the impact of HELP on STH infections

After the allocation of groups, there were no significant differences in the variables and characteristics between the intervention school and the control school, which indicates the similarity between different Orang Asli communities in terms of socioeconomic and epidemiological characteristics (Table 1).

**Table 1:** Baseline characteristics of the schoolchildren in the intervention (HELP) and control schools*

Characteristics	HELP	Control
N	172	145
Male/Female	82/90	73/72
Age (years)^a^	9 (8, 11)	9 (7, 11)
Fathers’ education level (at least 6 years)	106 (61.6)	77 (53.1)
Mothers’ education level (at least 6 years)	80 (46.5)	59 (40.7)
Working fathers	55 (32.0)	42 (29.0)
Working mothers	8 (4.7)	10 (6.9)
Low household income (<RM500)	112 (65.1)	99 (68.3)
Large family size (>7 members)	84 (48.8)	80 (55.2)
Piped water supply	86 (50.0)	83 (57.2)
Electricity	134 (77.9)	117 (80.7)
Presence of toilet in house	82 (47.7)	83 (57.2)
Trichuriasis	164 (95.3)	141 (97.2)
Ascariasis	90 (52.3)	73 (50.3)
Hookworm infection	63 (36.6)	45 (31.0)
Mixed STH infections	106 (62.7)	85 (59.0)

All values are number (%).

^a^Median (Interquartile range).

^*^No significant differences in the variables and characteristics between intervention and control schools.

### Impact of HELP on the STH reinfection rates

**Figure 3:**
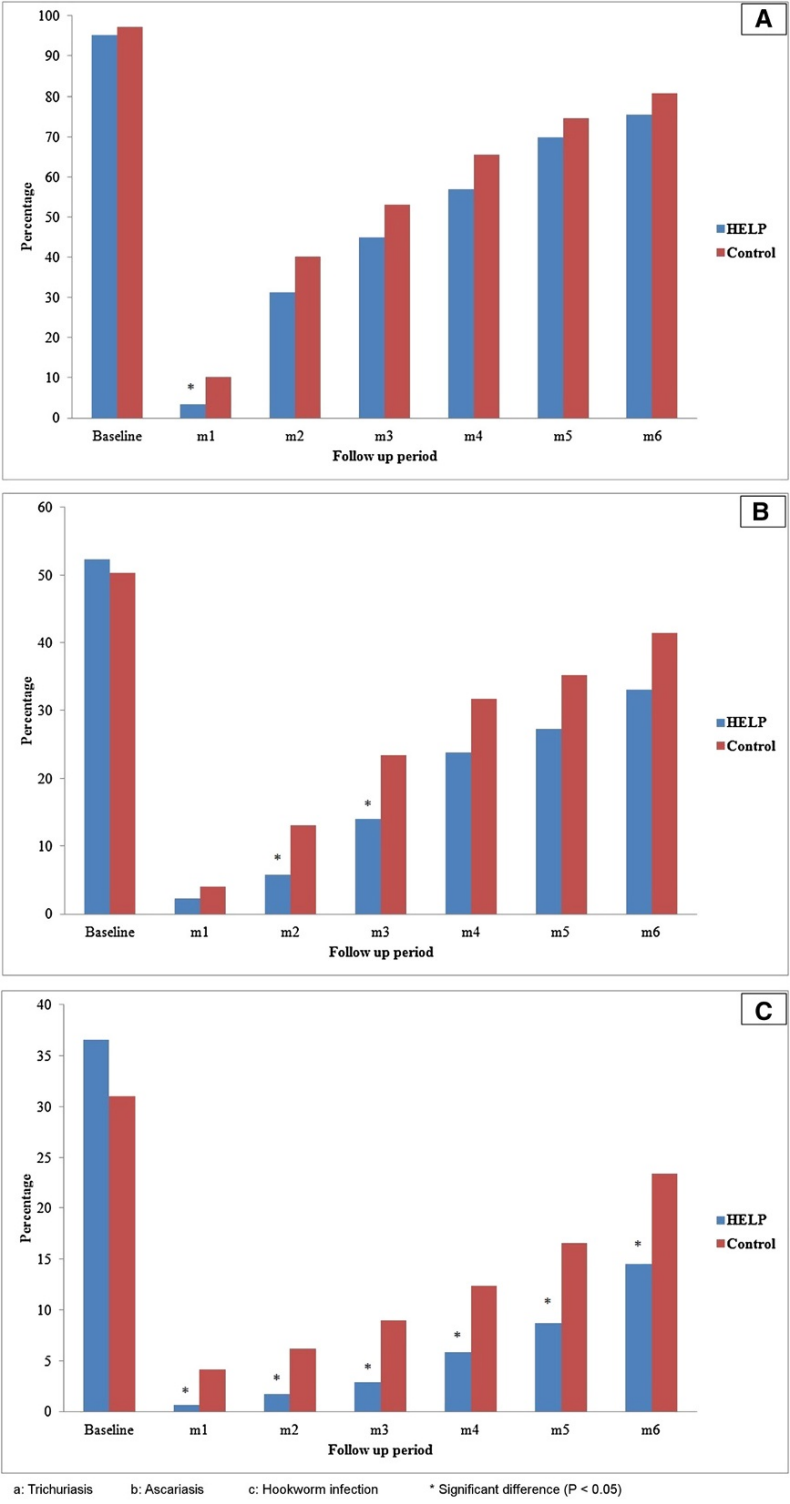
**Prevalence and incidence of STH infections among the intervention and control groups. A**: Trichuriasis. **B**: Ascariasis. **C**: Hookworm infection. *Significant difference (P < 0.05).

After a complete deworming the children were screened for the presence of STH infections monthly over the next 6 months. The baseline prevalence and incidence rates of trichuriasis, ascariasis and hookworm infections are shown in Figure 3 (a, b and c respectively). In Figure 3a, there was no significant difference in the incidence of trichuriasis between both groups. Similarly, Figure 3b shows that the incidence rates of ascariasis throughout the 6 months were lower in the HELP group compared to the control group, however the difference was only significant at the 2nd (5.8% vs 13.1%; P = 0.025) and 3rd month assessments (14.0% vs 23.4%; *P* = 0.029). Interestingly, Figure 3c shows that the incidence rates of hookworm infection throughout the 6 months assessment were significantly lower among children in the HELP group when compared to those in the control group (*P* < 0.05). It was found that the reinfection rates of trichuriasis and ascariasis after 6 months of complete deworming were almost 83.0%, 82.3% and 75.5% respectively among the control group, compared to 79.3%, 63.3% and 39.6% respectively among the HELP group.

### Impact of HELP on the STH reinfection intensity

**Figure 4:**
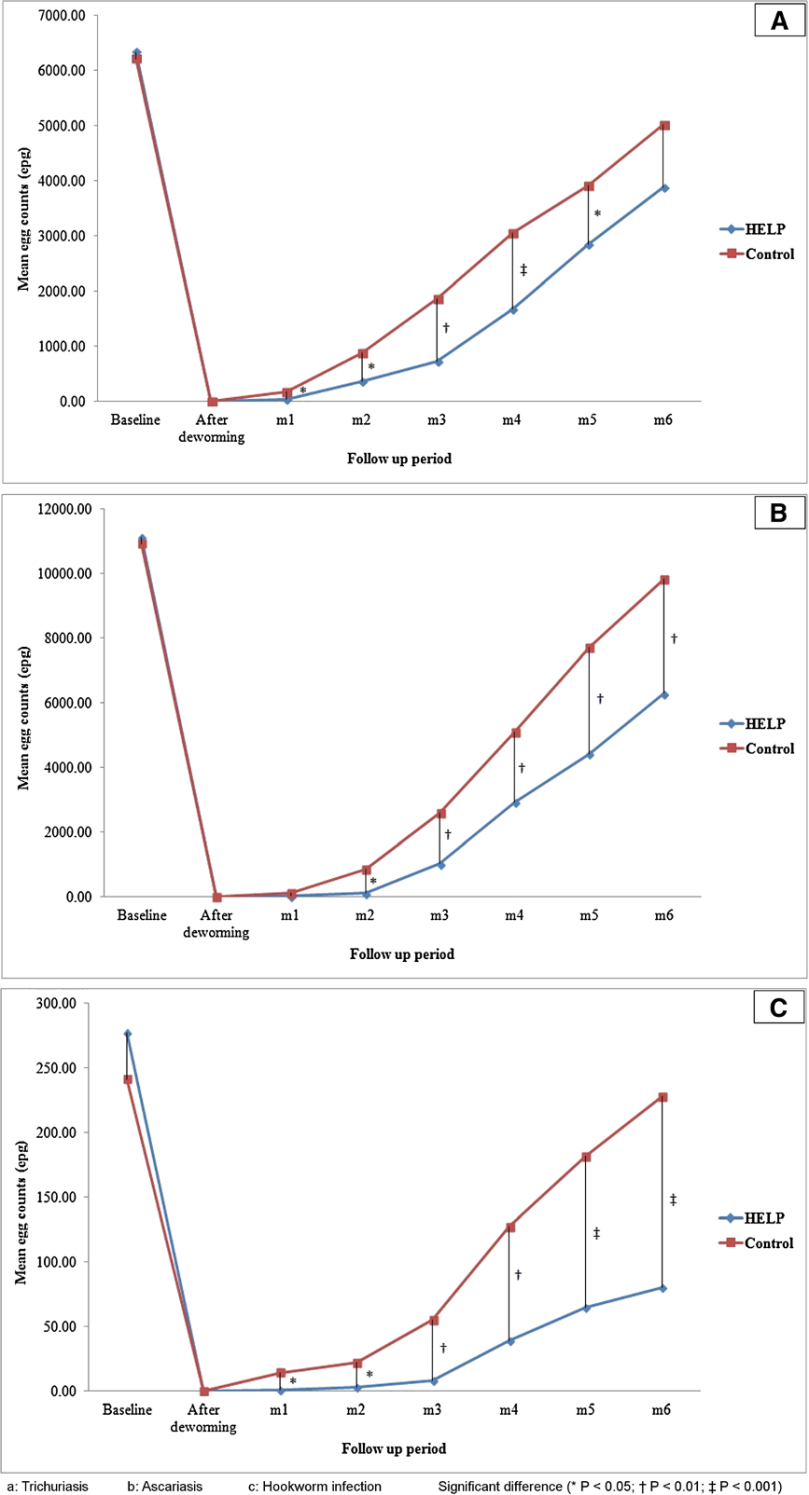
**Intensity of STH infections among the intervention and control groups. A**: Trichuriasis. **B**: Ascariasis. **C**: Hookworm infection. Significant difference (*P < 0.05; † *P* < 0.01; ‡ *P* < 0.001).

Figure 4 (a, b and c) shows the baseline and reinfection intensities of trichuriasis, ascariasis and hookworm infections respectively. It was found that HELP had a positive impact in protecting children from severe STH infections. The intensity of infections (of all three STH species) indicated by the egg counts (epg) was found to be significantly lower among children in the HELP group compared to those in the control group. After 6 months, the intensity of *Trichuris*, *Ascaris* and hookworm infections were reduced by 38.7%, 43.4% and 71.0% respectively among the intervention group, compared to 19.3%, 10.2% and 5.6% respectively among the control group. The significant difference was obvious with regards to hookworm infection throughout all assessments stages (*P* < 0.05). The intensity of trichuriasis was significantly lower in the HELP group compared to the control group, but the difference by month 6 was not statistically significant (*P* > 0.05).

### Impact of HELP on the KAP of Orang Asli towards STH infections

The KAP of Orang Asli people from both locations was assessed at baseline and 3 months after starting the intervention study and the results were presented in Tables 2 and 3. The participated children were belonging to 165 households (89 from Pos Betau and 76 from Kuala Koyan). At baseline, it was found that 117 respondents had heard about intestinal worm (62 from Pos Betau and 55 from Kuala Koyan). The results showed that knowledge about the role of dirty hands in the transmission of STH was significantly higher among participants from HELP group compared to those from control group (17.7% vs 5.5%; *P* = 0.041), while there was no significant difference in other variables of KAP towards STH infections between heads of households from both groups (*P* > 0.05).

**Table 2:** **Knowledge about intestinal helminths, symptoms, transmission and prevention among Orang Asli in the study area**

Variable	Baseline assessment				3 months assessment			
	HELP n (%)	Control n (%)	OR (95% CI)	*P*	HELP n (%)	Control n (%)	OR (95% CI)	*P*
Heard about intestinal worms	62 (69.7)	55 (72.4)	0.9 (0.4, 1.7)	0.703	80 (89.9)	59 (78.7)	1.6 (1.0, 2.7)	0.046
**Source of information**								
Clinic/hospitals	11 (17.5)	11 (20.1)	0.9 (0.6, 1.5)	0.755	15 (18.8)	16 (26.7)	0.8 (0.5, 1.2)	0.264
Mass media	2 (3.2)	2 (3.7)	0.9 (0.3, 2.5)	0.890*	2 (2.5)	2 (2.5)	0.9 (0.3, 2.3)	0.760*
School/schoolchildren	3 (4.8)	2 (3.7)	1.2 (0.4, 3.5)	0.762*	27 (33.3)	4 (6.8)	3.9 (1.5, 8.9)	< 0.001
Posters	0	0	NA	-	44 (55.0)	0 (0.0)	NA	< 0.001
Do not remember	28 (44.4)	22 (40.0)	1.1 (0.7, 1.6)	0.626	3 (3.8)	19 (32.2)	0.4 (0.3, 0.6)	< 0.001
**Type of worms**								
Roundworm	9 (14.3)	6 (10.9)	1.2 (0.6, 2.3)	0.583	26 (32.1)	8 (13.6)	2.0 (1.1, 3.9)	0.012
Pinworm	9 (14.5)	9 (16.4)	0.9(0.6, 1.5)	0.782	15 (18.8)	10 (16.9)	1.1 (0.6, 1.8)	0.785
Hookworm	0	0	NA	-	25 (30.9)	0 (0.0)	NA	< 0.001
Whipworm	0 (0.0)	1 (1.8)	NA	0.470	7 (8.6)	2 (3.4)	1.9 (0.6, 5.6)	0.211
**Signs and symptoms**								
Know at least one symptom	26 (41.9)	25 (45.5)	0.9 (0.6, 1.4)	0.702	60 (75.0)	33 (55.9)	1.6 (1.1, 2.3)	0.018
Abdominal pain	6 (9.5)	10 (18.2)	0.7 (0.5, 1.1)	0.171	30 (37.5)	16 (27.1)	1.3 (0.8, 2.1)	0.199
Abdominal distention	4 (6.5)	5 (9.1)	0.8 (0.4, 1.5)	0.733*	16 (20.0)	5 (8.5)	1.9 (0.9, 4.2)	0.061
Diarrhoea	9 (14.5)	9 (16.4)	0.9 (0.6 (1.5)	0.782	29 (35.8)	13 (22.0)	1.5 (0.9, 2.5)	0.079
Vomiting	3 (4.8)	3 (5.5)	0.9 (0.4, 2.1)	0.881*	8 (10.0)	3 (5.1)	1.6 (0.6, 4.2)	0.289
Loss of appetite	2 (3.2)	4 (7.3)	0.7 (0.4, 1.3)	0.481*	13 (16.2)	5 (8.5)	1.6 (0.7, 3.4)	0.177
Pale face	2 (3.2)	1 (1.8)	1.4 (0.3, 3.7)	0.831*	9 (11.1)	3 (5.1)	1.7 (0.6, 4.7)	0.208
Body weakness	6 (9.5)	6 (10.9)	0.9 (0.5, 1.7)	0.804	15 (18.8)	7 (11.9)	1.4 (0.7, 2.7)	0.272
Preanal itching	7 (11.1)	8 (14.8)	0.8 (0.5, 1.4)	0.550	12 (15.0)	12 (20.3)	0.8 (0.5, 1.3)	0.410
Blood in stool	2 (3.2)	1 (1.8)	1.4 (0.3, 7.1)	0.762*	3 (3.8)	1 (1.7)	1.7 (0.3, 9.4)	0.637*
Poor school performance	0	0	NA	-	20 (25.0)	1 (1.7)	10.3 (1.5, 51.7)	< 0.001
**Transmission**								
Know at least one way of transmission	24 (38.7)	20 (36.4)	1.1 (0.7, 1.6)	0.794	39 (48.8)	24 (40.0)	1.2 (0.8, 1.8)	0.303
Eating contaminated food	7 (11.3)	7 (12.7)	0.9 (0.5, 1.6)	0.811	16 (19.8)	7 (11.9)	1.5 (0.8, 2.8)	0.214
Dirty hands	11 (17.7)	3 (5.5)	2.3 (0.8, 6.5)	0.041	28 (33.8)	5 (8.5)	3.2 (1.4, 7.3)	< 0.001
Walking barefooted	8 (12.9)	3 (5.5)	1.8 (0.7, 4.8)	0.168	38 (46.9)	3 (5.1)	7.7 (2.6, 23.5)	< 0.001
Drinking untreated water	6 (9.7)	2 (3.6)	1.9 (0.6, 5.6)	0.196*	25 (30.9)	4 (6.7)	3.6 (1.4, 9.1)	< 0.001
Playing with soil	2 (3.2)	5 (9.1)	0.6 (0.4, 1.1)	0.251*	18 (22.5)	8 (13.3)	1.5 (0.8, 2.7)	0.168
Not cutting nails regularly	5 (7.9)	2 (3.6)	1.6 (0.5, 4.5)	0.447*	28 (34.6)	5 (8.5)	3.3 (1.5, 7.6)	< 0.001
Flies	0	0	NA	-	18 (22.5)	0 (0.0)	NA	< 0.001
**Prevention**								
Know at least one way for prevention	24 (38.7)	28 (50.9)	0.8 (0.5, 1.1)	0.185	52 (64.2)	27 (45.8)	1.5 (1.0, 2.3)	0.030
Taking de-worming drugs	10 (16.1)	17 (30.9)	0.7 (0.5, 0.9)	0.058	24 (30.0)	21 (35.6)	0.9 (0.6, 1.3)	0.486
Washing hands before eating	10 (16.1)	9 (16.4)	1.0 (0.6, 1.7)	0.973	45 (55.6)	13 (22.0)	2.5 (1.5, 4.1)	< 0.001
Wearing shoes when outside	5 (8.1)	2 (3.6)	1.7 (0.5, 5.5)	0.445*	31 (38.8)	6 (10.0)	3.2 (1.5, 6.9)	< 0.001
Boiling drinking water	4 (6.5)	4 (7.4)	0.9 (0.4, 1.9)	0.840*	19 (23.8)	9 (15.0)	1.4 (0.8, 2.5)	0.286
Washing vegetables before consumption	0	0	NA	-	25 (30.9)	0 (0.0)	NA	< 0.001
Cutting fingernails regularly	2 (3.2)	1 (1.8)	1.5 (0.4, 3.9)	0.762*	18 (22.5)	3 (5.1)	2.3 (1.4, 4.3)	0.004

OR, Odds ratio. CI, Confidence interval. NA, Not applicable due to small numbers.

*Fisher’s Exact test.

Significant association (*P* < 0.05).

**Table 3:** **Attitude and perceived practices towards intestinal helminths among Orang Asli in the study area**

Variable	Baseline assessment				3 months assessment			
	HELP n (%)	Control n (%)	OR (95% CI)	*P*	HELP n (%)	Control n (%)	OR (95% CI)	*P*
**Effects of intestinal helminths**								
Harmful to peoples’ health	36 (40.4%	26 (34.2)	1.2 (0.8, 1.9)	0.348	76 (85.4)	32 (42.1)	2.6 (1.9, 3.6)	< 0.001
Do not know	53 (59.6)	50 (65.8)	-	-	13 (14.6)	44 (57.9)	-	-
**Faeces as source of infections**								
Yes	24 (27.0)	28 (36.8)	0.7 (0.3, 1.7)	0.276	45 (50.6)	34 (44.7)	1.2 (0.7, 2.3)	0.620
No	1 (1.1)	2 (2.6)	-	-	1 (1.1)	2 (2.6)	-	-
Do not know	64 (71.9)	46 (60.5)	-	-	43 (48.3)	40 (52.6)	-	-
**Practices**								
Washing hand before eating	34 (38.2)	33 (43.4)	0.8 (0.4, 1.5)	0.469	80 (89.9)	38 (50.0)	3.5 (1.9, 6.4)	< 0.001
Washing hand after defecation	49 (55.1)	41 (53.9)	1.0 (0.6, 1.9)	0.887	73 (82.0)	43 (56.6)	3.5 (1.7, 7.1)	< 0.001
Washing hand with soap	16 (18.0)	18 (23.7)	0.7 (0.3, 1.5)	0.366	60 (67.4)	18 (23.7)	6.5 (3.2, 13.1)	< 0.001
Wearing shoes when outside	50 (56.2)	37 (48.7)	1.4 (0.7, 2.8)	0.336	81 (91.0)	41 (53.9)	8.6 (3.6, 20.3)	< 0.001
Cutting fingernails regularly	36 (40.4)	35 (46.1)	0.7 (0.5, 1.1)	0.469	68 (76.4)	46 (60.5)	2.1 (1.1, 4.1)	0.028
Washing vegetables before eating	33 (37.1)	31 (41.3)	0.8 (0.4, 1.6)	0.578	64 (71.9)	35 (46.7)	2.9 (1.5, 5.6)	0.001
Washing of fruits before eating	29 (32.6)	30 (39.5)	0.7 (0.4, 1.4)	0.357	53 (59.6)	34 (44.7)	1.8 (0.9, 3.3)	0.057
Boiling drinking water	21 (23.9)	17 (22.7)	1.0 (0.4, 2.6)	0.857	32 (36.4)	17 (22.7)	1.9 (0.9,3.9)	0.062
Indiscriminate defecation	58 (65.2)	51 (67.1)	0.9 (0.7, 1.4)	0.793	41 (46.1)	47 (61.8)	0.7 (0.5, 0.9)	0.043
Seeking treatment from clinic	88 (98.9)	71 (94.7)	1.2 (0.6, 2.7)	0.180	88 (100)	70 (93.3)	1.0 (0.5, 2.8)	0.094

OR, Odds ratio. CI, Confidence interval. NA, Not applicable due to small numbers.

Significant association (*P* < 0.05).

Three months after introducing HELP, the KAP regarding STH infections was reassessed among participants from both schools, with the results showing a significantly higher percentage of people who had heard about intestinal worms among the HELP group, with a notable increment from baseline assessment when compared to the control group (89.9% vs 78.7%; *P* = 0.046). Among them, the percentages of schoolchildren and posters acting as sources of information about STH were significantly higher among the HELP group when compared to the control. Overall, the knowledge of respondents in the HELP group regarding intestinal worms was obviously improved. The percentage of participants who knew at least one sign or symptom of STH infection was significantly higher in the HELP group compared to the control group (75.0% vs 55.9%; *P* = 0.018), with 25.0% of the respondents in the HELP group mentioning poor school performance compared to none in the control group (*P* < 0.001). Furthermore, knowledge surrounding the transmission and prevention of STH was significantly improved among the HELP group compared to the control group. In terms of improvements in the children’s understanding of STH, at the 3 month assessment only the HELP group mentioned posters as a source of information, hookworm as an example of worms, poor school performance as a symptom, flies as a way of transmission and washing vegetables before consumption as a preventive measure.

**Figure 5:**
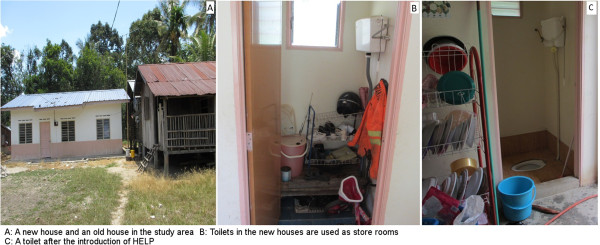
**New houses provided to Orang Asli people in some areas. A**: A new house and an old house in the study area. **B**: Toilets in the new houses are used as store rooms. **C**: A toilet after the introduction of HELP.

Interestingly, the practices of Orang Asli respondents in relation to STH infections were significantly improved among the intervention group when compared to the control group. A great improvement was noted in the percentage of those washing their hands before eating, wearing shoes when going outside the house, washing hands after defecation, and washing vegetables before consumption. A significant reduction in the percentage of those practicing open defecation was also reported among the intervention group compared to an unchanged percentage in the control group (*P* = 0.04). Likewise, it was observed that the use and cleanliness of toilets improved considerably among the intervention group (Figure 5A,B and C).

**Figure 6:**
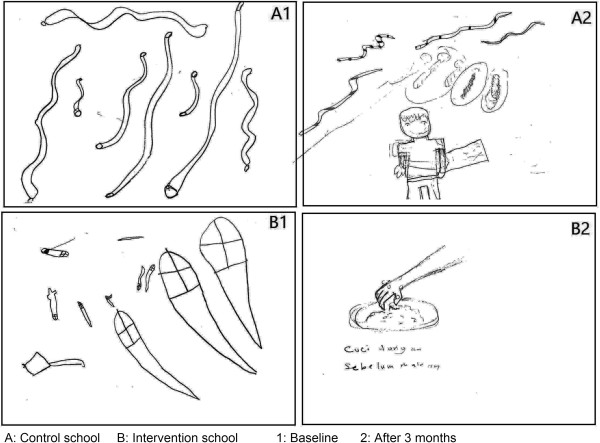
**Examples of drawing assessments for children at baseline and after 3 months. A**: Control school. **B**: Intervention school; 1: Baseline. 2: After 3 months.

As a part of the knowledge assessment, drawing activities were organized at both schools at baseline and after 3 months. At baseline, not all children were able to draw a clear or meaningful figure relating to intestinal worms. Subsequently, children from the intervention school showed a significant improvement with more than 75% of them being able to translate their understanding on intestinal worm morphology, modes of transmission and preventive measures in clear figures. On the other hand, children from the control school continued drawing unclear figures (Figure 6A and B).

### Impact of HELP on the knowledge of teachers about STH infections

The knowledge of teachers about intestinal helminth infections was assessed at baseline and after 3 months among 44 teachers from both schools (29 teachers from SKB and 15 from SKKK), and the results are shown in Table 4. At baseline, the results showed that the majority of the teachers from both schools had heard about intestinal worms, with there being no significant difference in their knowledge about signs, symptoms, transmission and prevention of intestinal helminth infections. About half of the teachers in both schools mentioned abdominal pain as a symptom of infection, not washing hands before eating acting as a mode of transmission, and taking deworming drugs as a preventive measure. That said, less than one third of them knew about any other points.

**Table 4:** **Knowledge about intestinal helminths, symptoms, transmission and prevention among teachers in both schools involved in the study**

Variable	Baseline assessment					3 months assessment		
	HELP n (%)	Control n (%)	OR (95% CI)	*P*	HELP n (%)	Control n (%)	OR (95% CI)	*P*
Heard about intestinal worms	25 (86.2)	13 (86.7)	0.9 (0.2, 5.9)	0.966*	29 (100)	14 (93.3)		0.341*
**Source of information**								
Clinic/hospitals	4 (16.0)	2 (15.4)	0.9 (0.2, 6.0)	0.961*	5 (17.2)	3 (21.4)	0.8 (0.3, 2.3)	0.741*
Internet	3 (12.0)	2 (15.4)	0.8 (0.3, 2.7)	0.770*	3 (10.3)	3 (21.4)	0.6 (0.2, 1.5)	0.373*
Mass media	4 (16.0)	2 (15.4)	1.2 (0.4, 4.3)	0.961*	3 (10.3)	2 (14.3)	0.8 (0.3, 2.5)	0.706*
School	0	0	NA	-	23 (79.3)	4 (28.6)	2.6 (1.0, 7.1)	< 0.001
**Type of worms**								
Roundworm	8 (32.0)	5 (38.5)	0.8 (0.3, 2.0)	0.690	27 (93.1)	5 (35.7)	5.2 (2.2, 12.2)	< 0.001
Pinworm	6 (24.0)	4 (30.8)	0.8 (0.3, 2.1)	0.709*	9 (31.0)	7 (50.0)	0.6 (0.3, 1.4)	0.228
Hookworm	9 (36.0)	6 (46.2)	0.7 (0.3, 1.8)	0.544	23 (79.3)	6 (42.9)	2.7 (1.2, 6.4)	0.035*
Whipworm	0	0	NA	-	11 (37.9)	0 (0.0)	NA	0.008*
**Signs and symptoms**								
Know at least one symptom	20 (80.0)	9 (69.2)	1.4 (0.6, 3.5)	0.459	28 (96.6)	9 (64.3)	3.4 (1.8, 6.7)	0.010*
Abdominal pain	14 (56.0)	7 (53.8)	1.1 (0.4, 2.5)	0.899	29 (100)	7 (50.0)	NA	< 0.001
Diarrhoea	10 (40.0)	7 (53.8)	0.7 (0.3, 1.7)	0.415	18 (62.1)	8 (57.1)	1.1 (0.5, 2.7)	0.757
Vomiting	6 (24.0)	3 (23.1)	1.0 (0.4, 2.9)	0.949*	6 (20.7)	4 (28.6)	0.8 (0.3, 1.9)	0.704
Loss of appetite	6 (24.0)	5 (38.5)	0.7 (0.3, 1.6)	0.457	6 (20.7)	4 (28.6)	0.8 (0.3, 1.9)	0.704*
Body weakness	7 (28.0)	2 (15.4)	1.7 (0.5, 6.3)	0.456*	7 (24.1)	4 (28.6)	0.9 (0.4, 2.2)	0.755
Blood in stool	4 (16.0)	2 (15.4)	1.0 (0.3, 3.5)	0.961*	1 (3.4)	1 (7.1)	0.6 (0.2, 2.7)	0.590*
Poor school performance	0	0	NA	-	12 (41.4)	1 (7.1)	5.6 (1.0, 38.7)	0.033*
**Transmission**								
Know at least one way of transmission	16 (64.0)	10 (76.9)	0.7 (0.2, 1.9)	0.486	29 (100)	11 (78.6)	NA	0.029*
Eating contaminated food	10 (40.0)	5 (38.5)	1.0 (0.4, 2.6)	0.927	17 (58.6)	8 (57.1)	1.0 (0.4, 2.5)	0.927
Dirty hands	13 (52.0)	8 (61.5)	0.8 (0.3, 1.9)	0.575	20 (69.0)	9 (64.3)	1.2 (0.5, 2.8)	0.795
Walking barefooted	7 (28.0)	4 (30.8)	0.9 (0.4, 2.4)	0.859	17 (58.6)	5 (35.7)	1.9 (0.8, 4.7)	0.159
Drinking untreated water	8 (32.0)	4 (30.8)	1.0 (0.4, 2.7)	0.938	5 (17.2)	5 (35.7)	0.5 (0.2, 1.3)	0.252
Playing with soil	2 (8.0)	1 (7.7)	1.0 (0.2, 4.5)	0.973*	20 (69.0)	4 (28.6)	3.1 (1.2, 8.5)	0.012
Not cutting nails regularly	5 (20.0)	4 (30.8)	0.7 (0.3, 1.7)	0.689*	10 (34.5)	6 (42.9)	0.8 (0.3, 1.8)	0.594
Flies	0	0	NA	-	11 (37.9)	1 (7.1)	5.0 (1.0, 34.3)	0.035*
**Prevention**								
Know at least one way for prevention	20 (80.0)	8 (61.5)	1.7 (0.7,4.1)	0.263*	29 (100)	10 (71.4)	NA	0.008*
Taking de-worming drugs	11 (44.0)	8 (61.5)	0.6 (0.2, 1.6)	0.305	17 (58.6)	7 (50.0)	1.3 (0.5, 2.9)	0.594
Washing hands before eating	8 (32.0)	5 (38.5)	0.8 (0.3, 2.0)	0.730*	29 (100)	9 (64.3)	NA	0.002*
Wearing shoes when outside	7 (28.0)	4 (30.8)	0.9 (0.4, 2.4)	0.858*	15 (51.7)	6 (42.9)	1.3 (0.5, 3.0)	0.586
Boiling drinking water	3 (12.0)	4 (30.8)	0.5 (0.2, 1.2)	0.203*	6 (20.7)	4 (28.6)	0.8 (0.3, 1.9)	0.704*
Washing vegetables before consumption	5 (20.0)	6 (46.2)	0.4 (0.2, 1.1)	0.135*	9 (31.0)	5 (35.7)	0.9 (0.4, 2.1)	0.759*

OR, Odds ratio. CI, Confidence interval. NA, Not applicable due to small numbers.

*Fisher’s Exact test.

Significant association (*P* < 0.05).

The follow-up assessment after 3 months showed a significant improvement in the knowledge of the teachers from the HELP school, at which time all of them (100%) mentioned at least one helminth type, one symptom, one way of transmission and one preventive measure for tackling intestinal helminth infections. On the other hand, the knowledge of teachers in the control school remained unchanged. Interestingly, 93.1%, 79.3% and 37.9% of the teacher in the HELP group mentioned roundworm, hookworm and whipworm, respectively, and these were significantly higher when compared with their counterparts from the control school. The results also showed that 41.4% and 37.9% of the teachers from the HELP school had mentioned poor school performance as a symptom for infections, as well as flies as a way of transmission and that these were significantly higher when compared to displays of such knowledge from the control group.

## Discussion

This study examined the impact of a developed health education learning package (HELP) on the incidence and intensity of STH infections, and on the STH-related knowledge of teachers and members of the Orang Asli communities. At baseline, we found that 98.7% of the participating children were infected by at least one STH species, with 96.2%, 51.4% and 34.1% of them being infected with *Trichuris*, *Ascaris* and hookworm respectively. Almost 71.9% and 29.0% of those afflicted with trichuriasis and ascariasis experienced moderate-to-heavy infections, while all hookworm infections were of light intensity. These findings are in agreement with the findings of previous studies conducted among Orang Asli communities in Malaysia [28–31, 34]. *Trichuris* infection is the predominant infection in Malaysia, which is in contrast with some previous reports from other Pacific and Southeast Asian countries such as Thailand and Vietnam where hookworms were reported as being the most prevalent parasitic species [43, 44], and China where ascariasis is predominant [12].

The results of the present study revealed that HELP was successful at reducing the intensity of STH infections. After 6 months, the intensity of hookworm infection and ascariasis were reduced by 70.0% and 43.4% respectively among the intervention group, compared to only 10.2% and 5.6% respectively among the control group. A smaller reduction was reported with trichuriasis, with a 38.7% reduction intensity being observed among the intervention group compared to 19.3% among the control group. Previous studies have found that health education has only a minimal, insignificant effect on hookworm infections [45, 46]. However, it should be noted that in the underprivileged communities there are several barriers to affecting positive change beyond the scope of simply providing education. One such issue is that the option for children to consistently wear shoes may be limited due to the lack of familial financial resources, meaning that people are unable to purchase more than one pair of shoes, and in some cases may not even be able to afford shoes for all their family members [47, 48]. Similarly, we observed that most Orang Asli children walked and played barefooted. Upon investigation, we found that most of these children had only school shoes and their parents did not allow them to use these shoes when moving around their villages. In order to alleviate this issue, HELP’s aid kit contains slippers, hand soap and nail clippers in order to help the children practice what they have learned. Although the prevalence of *Ascaris* (at 6 months) and *Trichuris* infections were lower among children from the intervention school compared to those from the control school, the differences were not statistically significant. This could be due to the easier mode of transmission enjoyed by these parasites (ingestion of infective eggs in contaminated food or water, or transference from contaminated fingers) when compared with hookworm. In such a heavily contaminated environment, it seemed that acquiring these infections can hardly be avoided. On the other hand, HELP’s significant impact in reducing infections intensity is consistent with the WHO’s control strategy, which aims to curtail parasitic transmission dynamics and thus reduce the worm burden of these infections (elimination of morbidity, not parasites), due to the total eradication of STH infections proving to be impossible in the regions rural communities at this time [34, 49].

Previous studies showed that sanitation, with or without health and hygiene education, reduces the prevalence and intensity of STH infections, the impact of which was further improved when combined with deworming [20, 50]. However, improving sanitation in highly endemic communities may not attain the desired impact without a parallel improvement in hygiene and health-related behaviours amongst the targeted population [51]. In Malaysia, the government has made intensive efforts to improve the quality of life of indigenous people throughout the country, with their main strategy being to reallocate those living in remote areas to new settlements at the periphery of towns. However, the adherence of the Orang Asli people in Peninsular Malaysia to their jungle habitats has constrained these efforts. According to the annual report of the Ministry of Rural and Regional Development, hundreds of houses were built or restored for Orang Asli people [52]. During our survey, we observed that the toilets were used as storage rooms due to cultural beliefs that toilets should not be located inside the house and also the lack of knowledge about the impact.

Few studies have previously investigated the impact of health education intervention on STH prevalence rates and intensities [45, 53–57]. A previous national project was carried out in the Seychelles over a two year period with the aim being to increase public awareness and provide information about intestinal parasitic infection (IPI) control using printed materials (newspapers, posters, leaflets) and electronic media (radio, television, audiovisual aids) [54]. The project achieved a 44% reduction in the prevalence of IPI, whilst the intensity of *Trichuris* infection, the predominant parasite, was reduced by 50% (from 780 to 370 eggs per g of faeces).

A previous study in the Peruvian Amazon investigated the impact of a school-based health hygiene education programme, which showed that after four months there was a significant reduction in *Ascaris* infection intensity among children from intervention schools when compared with children from control schools, though the differences in *Trichuris* and hookworm infections were not significant [45]. Interestingly, a recent study among Chinese children reported that the incidence of STH infections (ascariasis and trichuriasis) proved to be 50% lower in the intervention group who received a health education package involved a teacher-training workshop, as well as a cartoon based video, pamphlets, posters, and drawing and essay competitions compared to the control group who only received posters [57]. By contrast, a previous study in Jakarta, Indonesia, found no significant differences in the prevalence of *Ascaris* infection between children who received health education intervention over the course of 5 months when compared to their counterparts in the control group [55]. Similarly, Aung et al. [53] found no significant impact for hand washing intervention among children in Rangoon, Burma.

Our findings showed that HELP significantly improved the KAP of Orang Asli people towards STH infections. At the 3 month assessment point, a significant percentage of respondents mentioned hookworms, the effects of STH on school performance, the role of flies in transmission and washing vegetables before consumption as a preventive measure compared to none at the baseline assessment. Moreover, significant improvements were also reported with other variables (e.g. roundworms, transmission by contaminated hands and walking barefooted, and prevention by wearing shoes when outside the house and washing hands before eating). These findings were in agreement with previous studies elsewhere [45, 57]. Bieri et al. [57] showed a significant impact in increasing the KAP among their intervention group. For instance, the rate of hand washing increased from 46.0% to 98.9% among the intervention group whilst remaining unchanged in the control group (from 54.0% to 54.2%). Similarly, almost twice as many intervention children (63.3% *vs* 33.4%) reported washing their hands after defecating.

The present study shows that the rates of hand washing before eating and after defecation, wearing shoes when outside and hand washing with soap were almost doubled among the intervention group compared to unchanged rates among the control group. We observed children wearing their slippers while they were playing or walking outside, with a smaller (yet still significant) percentage of participants making good use of the distributed nail clippers and soaps. Furthermore, our findings showed that the knowledge of the teachers in the intervention school was significantly improved upon when compared to the control school. This was imperative to ensure an effective contribution of teachers when it came to implementing follow up HELP activities with the children.

Although the present study is the first to develop a health education package against STH infections in Malaysia, some limitations related to our study design (open-label controlled trial) and methods should be considered when interpreting the study’s findings. The comparison of two schools only might be not enough to rule out that the significant lower prevalence and intensity reported among the intervention group were not due to differences in the STH status between the two areas (a cluster randomized controlled trial is required). However, the improvements in the knowledge and practices of children and people in this group were obvious and therefore explain most of this significant impact. Moreover, previous studies among these children found that the prevalence and intensity of STH in both areas were similar at baseline and at 6 month after complete deworming [31, 33, 34]. Furthermore, the high incidence rates of *Ascaris* and *Trichuris* reported in both groups by 6 months may indicate the similar STH status in both areas and support that HELP was effective in reducing the exposure to these parasites and thereby reducing the intensity of infections and the incidence of hookworm as well. Anthelmintic treatment was not distributed to preschool children and adult individuals; however they still serve as a source of infections in these communities. In order to reduce transmission and achieve sustainable control, deworming should be extended to these age groups as well [58].

### Pros and cons of HELP

This health education learning package (HELP) was developed and introduced to Orang Asli schoolchildren as the first health education programme among these indigenous populations. The package proved effective among these children, particularly in terms of reducing the morbidity of STH infections. The package may also be useful in controlling other intestinal parasites, as the health messages cover preventative messages that can also apply to such parasites. HELP messages are restricted to helping improve personal hygiene practices, while the other factors such as poverty and low levels of education require more direct intervention by the government. It is recommended that interventions such as periodic deworming, providing provision of safe drinking water and providing toilets within rural households should be seriously considered by the government.

That said, the target population should also find alternative ways to help themselves and care for the health of their children. Such methods could include boiling drinking water before consumption, not defecating openly in the vicinity of house or village living areas instead defecating over a green leaf and then burying the stool. School-based programmes could prove to be particularly cost effective, as schools already have an available and sustained infrastructure with a skilled workforce that has a close relationship with the schoolchildren and the community. Due to the cost-effectiveness of school-based implementation, HELP should cost as little as RM5 (1.5 USD) per child including the slippers, hand soap and nails clipper. This cost could be further reduced with bigger wholesale orders for HELP items. The main components of the package (posters, comic book, video songs and the puppets and theatre) can be produced extremely cheaply to be used at the schools.

Besides HELP, our recommendation would be that anthelmintics drugs (albendazole tablets) should be distributed twice a year, at the beginning of school semesters. Such drugs are affordable, costing just a few pennies, and often provided for free from different agencies as a donation to rural populations. The involvement of teachers in such a programme is perceived as being crucial in order to achieve the sustainability and efficiency of the control programme. HELP is a school-based package, therefore its activities may disturb other forms of schooling and may add a burden to the teachers. However, this could be overcome by improving and integrating the package in to the overall curriculum and normal school activities. The benefits of school-based periodic deworming programmes are likely to be enhanced when a sustained health hygiene education intervention programme is integrated into school curricula. Nevertheless, fusing or integrating health education with other subjects such as science requires careful planning across subjects and levels. A good option would be for HELP to be integrated with the existing Doktor Muda (Young Doctor) programme [59], which would then be fully implemented in schools serving Orang Asli and other indigenous communities.

## Conclusion

Soil-transmitted helminths are highly prevalent among Orang Asli children in rural Malaysia, with the trend of infections remaining largely unchanged since the 1920s. This supports an urgent need to start an integrated and effective STH control programme. A school-based health education learning package (HELP) was developed and evaluated among Orang Asli schoolchildren in Lipis, Pahang. The findings showed that the package had a significant impact in reducing the intensity of all three main STH infections and in the prevalence of hookworm infections as a whole. Furthermore, the knowledge of both teachers and children’s parents about STH was significantly improved upon, factors which are imperative to encourage community mobilization that will enhance prevention and instil better knowledge on the transmission and prevention of these infections.

## Electronic supplementary material

Additional file 1: **The three posters for the health messages.** (PDF 1 MB)

Additional file 2: **The comic book used by this study.** (PDF 11 MB)

## References

[1] Hotez PJ, Kamath A (2009). Neglected tropical diseases in sub-Saharan Africa: review of their prevalence, distribution, and disease burden. PLoS Negl Trop Dis.

[2] World Health Organization (2002). WHO Technical Series Report, 912. Prevention and control of schistosomiasis and soil-transmitted helminthiasis.

[3] World Health Organization (2006). Schistosomiasis and soil-transmitted helminth infections – preliminary estimates of the number of children treated with albendazole or mebendazole. Wkly Epidemiol Rec.

[4] Pullan L, Brooker SJ (2012). The global limits and population at risk of soil-transmitted helminth infections in 2010. Parasit Vectors.

[5] Nokes C, Bundy DA (1994). Does helminth infection affect mental processing and educational achievement?. Parasitol Today.

[6] Ahmed A, Al-Mekhlafi H, Azam M, Ithoi I, Al-Adhroey A, Abdulsalam A, Surin J (2012). Soil-transmitted helminthiasis: a critical but neglected factor influencing school participation of Aboriginal children in rural Malaysia. Parasitol.

[7] Al-Mekhlafi HMS, Azlin M, NorAini U, Shaik A, Sa'aih A, Fatmah A, Ismail MG, Ahmad Firdaus MS, Aisah MY, Rozlida AR, Moktar N (2005). Malnutrition and soil-transmitted helminthiasis among Orang Asli children in Selangor, Malaysia. Asia Pac J Clin Nutr.

[8] Mahalanabis D, Simpson TW, Bhattacharjee AK (1979). Malabsorption of water miscible vitamin A in children with giardiasis and ascariasis. Am J Clin Nutr.

[9] Bleakley H (2007). Disease and development: evidence from hookworm eradication in the American South. Quart J Econom.

[10] World Health Organization (2005). Deworming for health and development. Report of the third global meeting of the partners for parasite control.

[11] Hong ST, Chai JY, Choi MH, Huh S, Rim HJ, Lee SH (2006). A successful experience of soil-transmitted helminths control in the Republic of Korea. Korean J Parasitol.

[12] Yap P, Du ZW, Chen R, Zhang LP, Wu FW, Wang J, Wang XZ, Zhou H, Zhou XN, Utzinger J, Steinmann P (2012). Soil-transmitted helminth infections and physical fitness in school-aged Bulang children in southwest China: results from a cross-sectional survey. Parasit Vectors.

[13] Kobayashi A, Hara T, Kajima J (2006). Historical aspects for the control of soil-transmitted helminthiases. Parasitol Int.

[14] World Health Organization (2012). Eliminating Soil-Transmitted Helminthiases as a Public Health Problem in Children.

[15] Jia T-W, Melville S, Utzinger J, King CH, Zhou X-N (2012). Soil-transmitted helminth reinfection after drug treatment: A systematic review and meta-analysis. PLOS Negl Trop Dis.

[16] Campbell SJ, Savage GB, Gray DJ, Atkinson J-AM, Soares Magalhães RJ, Nery SV, McCarthy JS, Velleman Y, Wicken JH, Traub RJ, Williams JM, Andrews RM, Clements ACA (2014). Water, Sanitation, and Hygiene (WASH): A Critical Component for Sustainable Soil-Transmitted Helminth and Schistosomiasis Control. PLoS Negl Trop Dis.

[17] Haswell-Elkins M, Elkins DB, Manjula K, Michael E, Anderson RM (1988). An investigation of hookworm infection and reinfection following mass anthelmintic treatment in the south Indian fishing community of Vairavankuppam. Parasitol.

[18] Vercruysse J, Albonico M, Behnke JM, Kotze AC, Prichard RK, McCarthy JS, Montresor A, Levecke B (2011). Is anthelmintic resistance a concern for the control of human soil-transmitted helminths?. Int J Parasitol Drugs Drug Resist.

[19] Diawara A, Halpenny CM, Churcher TS, Mwandawiro C, Kihara J, Kaplan RM, Streit TG, Idaghdour Y, Scott MY, Basáñez M-G, Prichard RK (2013). Association between response to albendazole treatment and β-tubulin genotype frequencies in soil-transmitted helminths. PLoS Negl Trop Dis.

[20] Asaolu SO, Ofoezie IE (2003). The role of health education and sanitation in the control of helminth infections. Acta Trop.

[21] Hotez PJ, Bundy DAP, Beegle K, Brooker S, Drake L, de Silva N, Montresor A, Engels D, Jukes M, Chitsulo L, Chow J, Laxminarayan R, Michaud C, Bethony J, Correa-Oliveira R, Shuhua X, Fenwick A, Savioli L, Jamison DT, Breman JG, Measham AR, Alleyne G, Claeson M, Evans DB, Jha P, Mills A, Musgrove P (2006). Helminth Infections: Soil-transmitted Helminth Infections and Schistosomiasis. Disease Control Priorities in Developing Countries.

[22] Mahmood AA, Khairul Anuar A, Sidik K, Salmah I, Suzainur KA, Mohammed NHM, Irwan H (2002). Comparison of prevalence of intestinal parasitic infections in school children in Kuala Lumpur. J Univ Malaya Med Centre.

[23] Jamaiah I, Rohela M (2005). Prevalence of intestinal parasites among members of the public in Kuala Lumpur, Malaysia. Southeast Asian J Trop Med Public Health.

[24] Russell PF (1928). Nematodes in man in the straits settlements: A preliminary report. Medical J Malaysia.

[25] Kan SP (1982). Soil transmitted helminthiases in Selangor. Malaysia. Med J Malaysia.

[26] Bundy DAP, Kan SP, Rose R (1988). Age-related prevalence, intensity and frequency distribution of gastrointestinal helminth infection in urban slum children from Kuala Lumpur, Malaysia. Trans R Soc Trop Med Hyg.

[27] Dunn FL (1972). Intestinal parasitism in Malayan aborigines (Orang Asli). Bull World Health Organ.

[28] Norhayati M, Oothuman P, Azizi O, Fatmah MS (1997). Efficacy of single dose albendazole on the prevalence and intensity of infection of soil-transmitted helminths in Orang Asli children in Malaysia. Southeast Asian J Trop Med Public Health.

[29] Al-Mekhlafi MS, Atiya AS, Lim YA, Mahdy AK, Ariffin WA, Abdullah HC, Surin J (2007). An unceasing problem: soil-transmitted helminthiases in rural Malaysian communities. Southeast Asian J Trop Med Public Health.

[30] Ngui R, Ishak S, Chuen CS, Mahmud R, Lim YAL (2011). Prevalence and risk factors of intestinal parasitism in rural and remote West Malaysia. PLoS Negl Trop Dis.

[31] Nasr NA, Al-Mekhlafi HM, Ahmed A, Roslan MA, Bulgiba A (2013). Towards an effective control programme of soil-transmitted helminth infections among Orang Asli in rural Malaysia. Part 1: Prevalence and associated key factors. Parasit Vectors.

[32] Nasr NA, Al-Mekhlafi HM, Ahmed A, Roslan MA, Bulgiba A (2013). Towards an effective control programme of soil-transmitted helminth infections among Orang Asli in rural Malaysia. Part 2: Knowledge, attitude, and practices. Parasit Vectors.

[33] Hesham Al-Mekhlafi M, Surin J, Atiya AS, Ariffin WAW, Mahdy AKM, Abdullah HC (2008). Pattern and predictors of soil-transmitted helminth re-infection among aboriginal school children in rural peninsular Malaysia. Acta Trop.

[34] Ahmed A, Al-Mekhlafi HM, Choy SH, Ithoi I, Al-Adhroey AH, Abdulsalam AM, Surin J (2011). The burden of moderate-to-heavy soil-transmitted helminth infections among rural Malaysian aborigines: an urgent need for an integrated control programme. Parasit Vectors.

[35] Green LW (1974). Toward cost-benefit evaluations of health education: some concepts, methods, and examples. Health Education Monographs.

[36] Anuar TS, Salleh FM, Moktar N (2014). Soil-transmitted helminth infections and associated risk factors in three Orang Asli tribes in Peninsular Malaysia. Sci Rep.

[37] Thyssen PJ, Moretti TC, Ueta MT, Ribeiro OB (2004). The role of insects (Blattodea, Diptera and Hymenoptera) as possible mechanical vectors of helminths in the domiciliary and peridomiciliary environment. Cadernos de Saúde Pública.

[38] Sulaiman S, Sohadi AR, Yunus H, Iberahim R (1988). The role of some Cyclorrhaphan flies as carriers of human helminths in Malaysia. Med Vet Entomol.

[39] Jozefzoon LME, Oostburg BFJ (1994). Detection of hookworm and hookworm-like larvae in human fecocultures in Suriname. Am J Trop Med Hyg.

[40] Montresor A (2007). Arithmetic or geometric means of eggs per gram are not appropriate indicators to estimate the impact of control measures in helminth infections. Trans R Soc Trop Med Hyg.

[41] Albonico M, Rinaldi L, Sciascia S, Morgoglione ME, Piemonte M, Maurelli MP, Musella V, Utzinger J, Ali SM, Ame SM, Cringoli G (2013). Comparison of three copromicroscopic methods to assess albendazole efficacy against soil-transmitted helminth infections in school-aged children on Pemba Island. Trans R Soc Trop Med Hyg.

[42] Olsen A, Nawiri J, Friis H (2000). The impact of iron supplementation on reinfection with intestinal helminths and *Schistosoma mansoni* in western Kenya. Trans R Soc Trop Med Hyg.

[43] Anantaphruti MT, Waikagul J, Maipanich W, Nuamtanong S, Pubampen S (2004). Soil-transmitted helminthiases and health behaviors among schoolchildren and community members in a west-central border area of Thailand. Southeast Asian J Trop Med Public Health.

[44] Yajima A, Jouquet P, Do TD, Dang TC, Tran CD, Orange D, Montresor A (2009). High latrine coverage is not reducing the prevalence of soil-transmitted helminthiasis in Hoa Binh province, Vietnam. Trans R Soc Trop Med Hyg.

[45] Gyorkos TW, Maheu-Giroux M, Blouin B, Casapia M (2013). Impact of health education on soil-transmitted helminth infections in schoolchildren of the Peruvian Amazon: a cluster-randomized controlled trial. PLoS Negl Trop Dis.

[46] Brooker S, Bethony J, Hotez PJ (2004). Human hookworm infection in the 21st century. Adv Parasitol.

[47] Mascarini-Serra L (2011). Prevention of Soil-transmitted Helminth Infection. J Glob Infect Dis.

[48] Ayode D, McBride CM, de Heer HD, Watanabe E, Gebreyesus T, Tora A, Tadele G, Davey G (2013). A qualitative study exploring barriers related to use of footwear in rural highland Ethiopia: Implications for neglected tropical disease control. PLoS Negl Trop Dis.

[49] World Health Organisation (2012). Soil-transmitted helminthiases. Eliminating soil-transmitted helminthiases as a public health problem in children: Progress report 2001–2010 and strategic plan 2011–2020.

[50] Hawdon JM (2014). Controlling soil-transmitted helminths: time to think inside the box?. J Parasitol.

[51] Sow S, de Vlas SJ, Polman K, Gryseels B (2004). Hygiene practices and contamination risks of surface waters by schistosome eggs: the case of an infested village in Northern Senegal. Bull Soc Pathol Exot.

[52] Ministry of Rural and Regional Development (2010). Improving Rural Basic Infrastructure. Annual Report.

[53] Aung Myo H, Thein H, Myat Lay K, Than Saw (1988). Hand washing intervention to reduce ascariasis in children. Trans R Soc Trop Med Hyg.

[54] Albonico M, Shamlaye N, Shamlaye C, Savioli L (1996). Control of intestinal parasitic infections in Seychelles: a comprehensive and sustainable approach. Bull World Health Organization.

[55] Hadidjaja P, Bonang E, Suyardi MA, Abidin SAN, Ismid IS, Margono SS (1998). The effect of intervention methods on nutritional status and cognitive function of primary school children infected with *Ascaris lumbricoides*. Am J Trop Med Hyg.

[56] Albright JW, Basaric-Keys J (2006). Instruction in behavior modification can significantly alter soil-transmitted helminth (STH) re-infection following therapeutic de-worming. Southeast Asian J Trop Med Public Health.

[57] Bieri FA, Gray DJ, Williams GM, Raso G, Li YS, Yuan L, He Y, Li RS, Guo FY, Li SM, McManus DP (2013). Health-education package to prevent worm infections in Chinese schoolchildren. N Engl J Med.

[58] Truscott JE, Hollingsworth TD, Brooker SJ, Anderson RM (2014). Can chemotherapy alone eliminate the transmission of soil transmitted helminths?. Parasit Vectors.

[59] Jaafar N, Omar K, Ahmad J, Wan Hussein WS, MANAF ZA: **The “Doktor Muda” health promotion programme: A process evaluation. Accessed on 4 June 2014.** http://www.seameo.org/vl/library/dlwelcome/projects/jasper/jasper05/jasper05.htm

