# Supplementary material for: Developing and evaluating health education learning package (HELP) to control soil-transmitted helminth infections among Orang Asli children in Malaysia
Source: Parasit Vectors. 2014 Sep 2;7:416. doi: 10.1186/1756-3305-7-416 (PMC4261692; doi:10.1186/1756-3305-7-416)
Supplement: Supplementary file 1 — Additional file 1: The three posters for the health messages. (PDF 1 MB) [file 13071_2014_1598_MOESM1_ESM.pdf]

Basuhlah tangan

- ☑ sebelum makan.
- ☑ selepas bermain di tanah.
- ☑ selepas ke tandas.

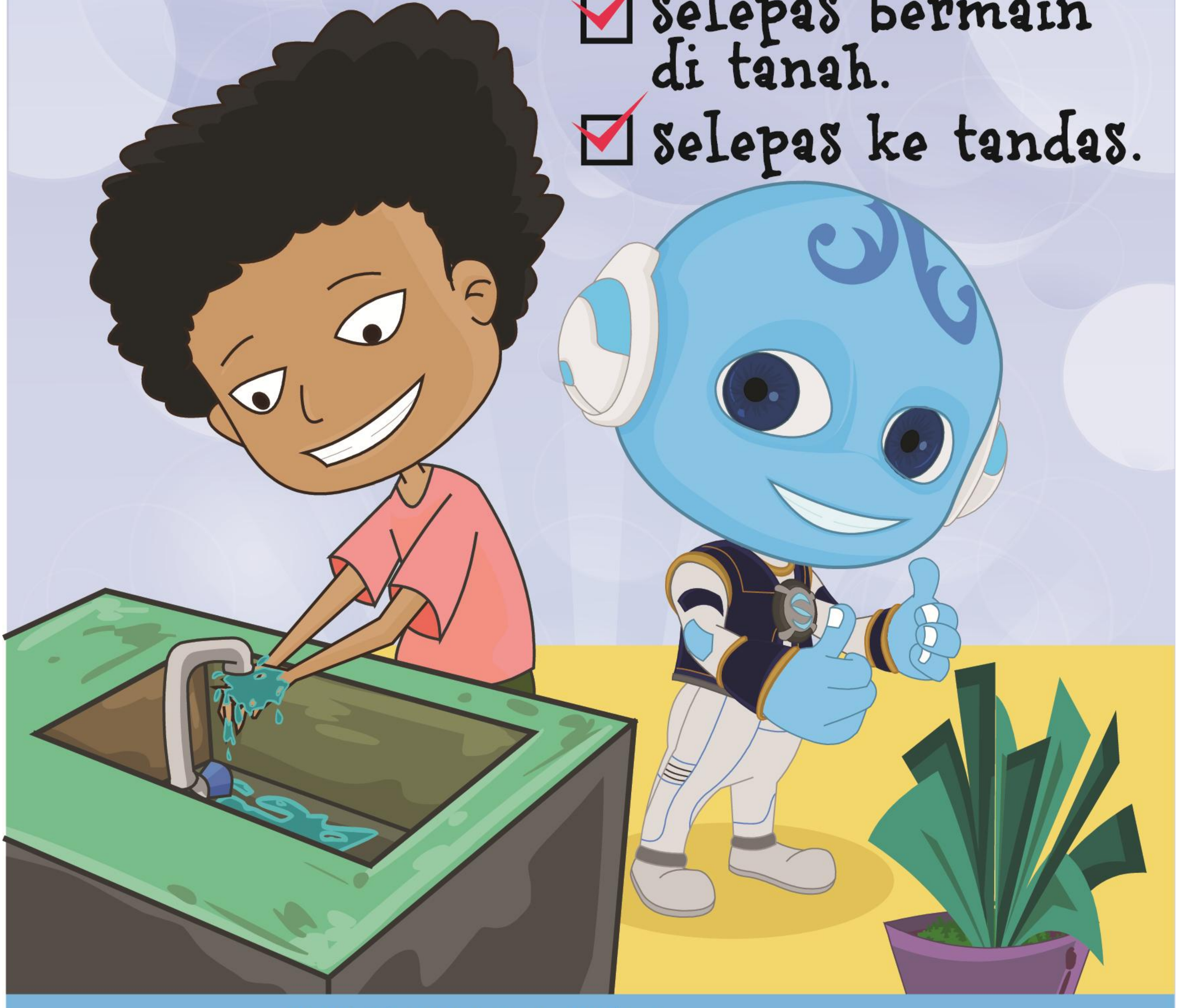

Health Education Learning Package
to control Soil-Transmitted Helminthiasis among Orang Asli Schoolchildren

Project team: Assoc. Prof. Dr. Hesham M. Al-Mekhlafi
Professor Dr. Rohela Mahmud
Assoc. Prof. Dr. Yvonne Al Lim
Dr. Ahmed K. Al-Delaimy

Department of Parasitology, Faculty of Medicine, University of Malaya

Contact: halmekhlafi@yahoo.com, halmekhlafi@um.edu.my

UNIVERSITI MALAYA
The Leader in Research & Innovation

In collaboration with:
JAKOA
JABATAN KEMAJUAN ORANG ASLI
animagis sdn bhd

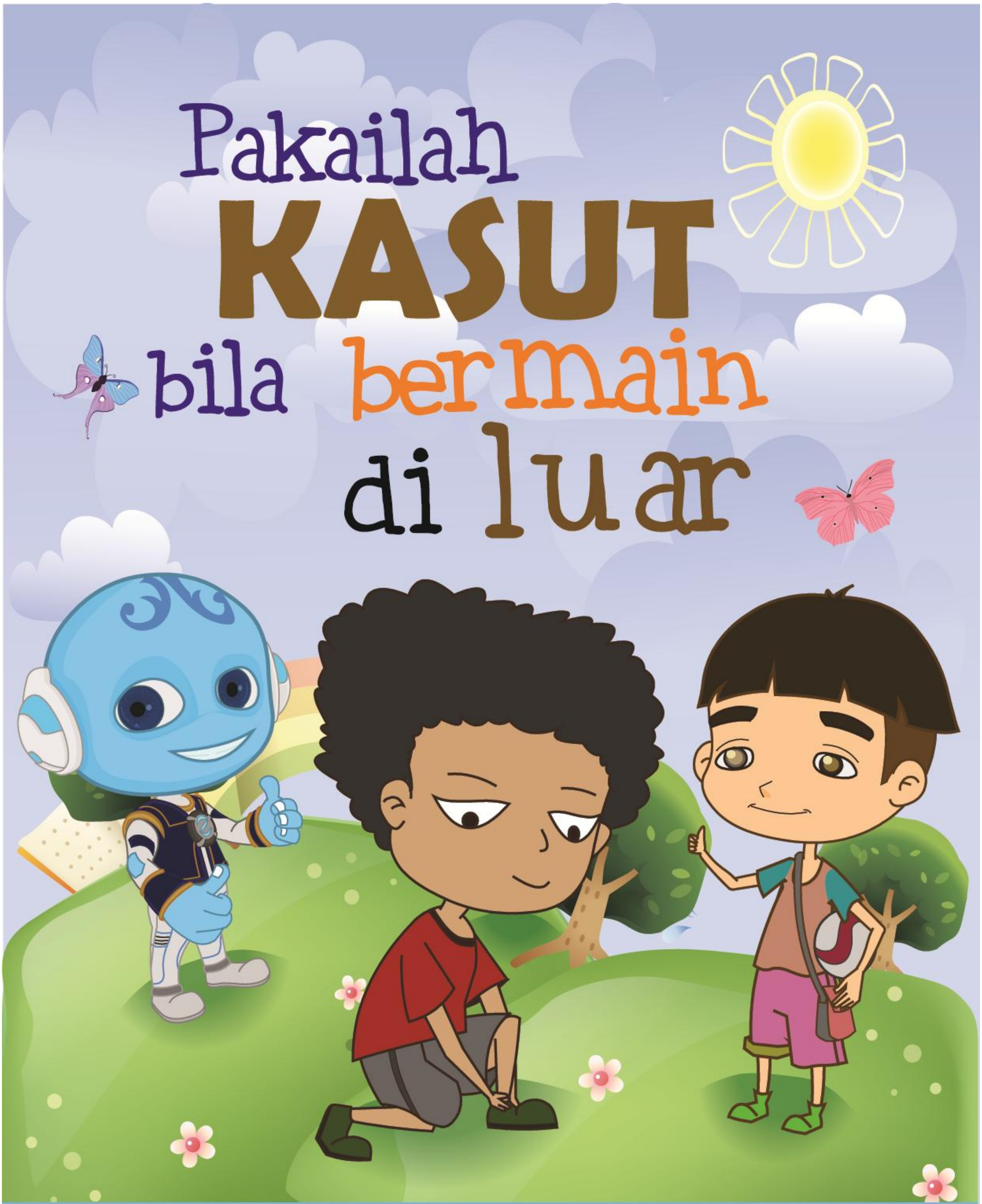

Health Education Learning Package
to control Soil-Transmitted Helminthiasis among Orang Asli Schoolchildren

Project team: Assoc. Prof. Dr. Hesham M. Al-Mekhlafi
Professor Dr. Rohela Mahmud
Assoc. Prof. Dr. Yvonne Ai Lim
Dr. Ahmed K. Al-Delaimy

Department of Parasitology, Faculty of Medicine, University of Malaya

Contact: halmekhlafi@yahoo.com, halmekhlafi@um.edu.my

UNIVERSITI MALAYA
The Leader in Research & Innovation

In collaboration with:

JAKOA
JABATAN KEMAJUAN ORANG ASLI

animagis sdn bhd

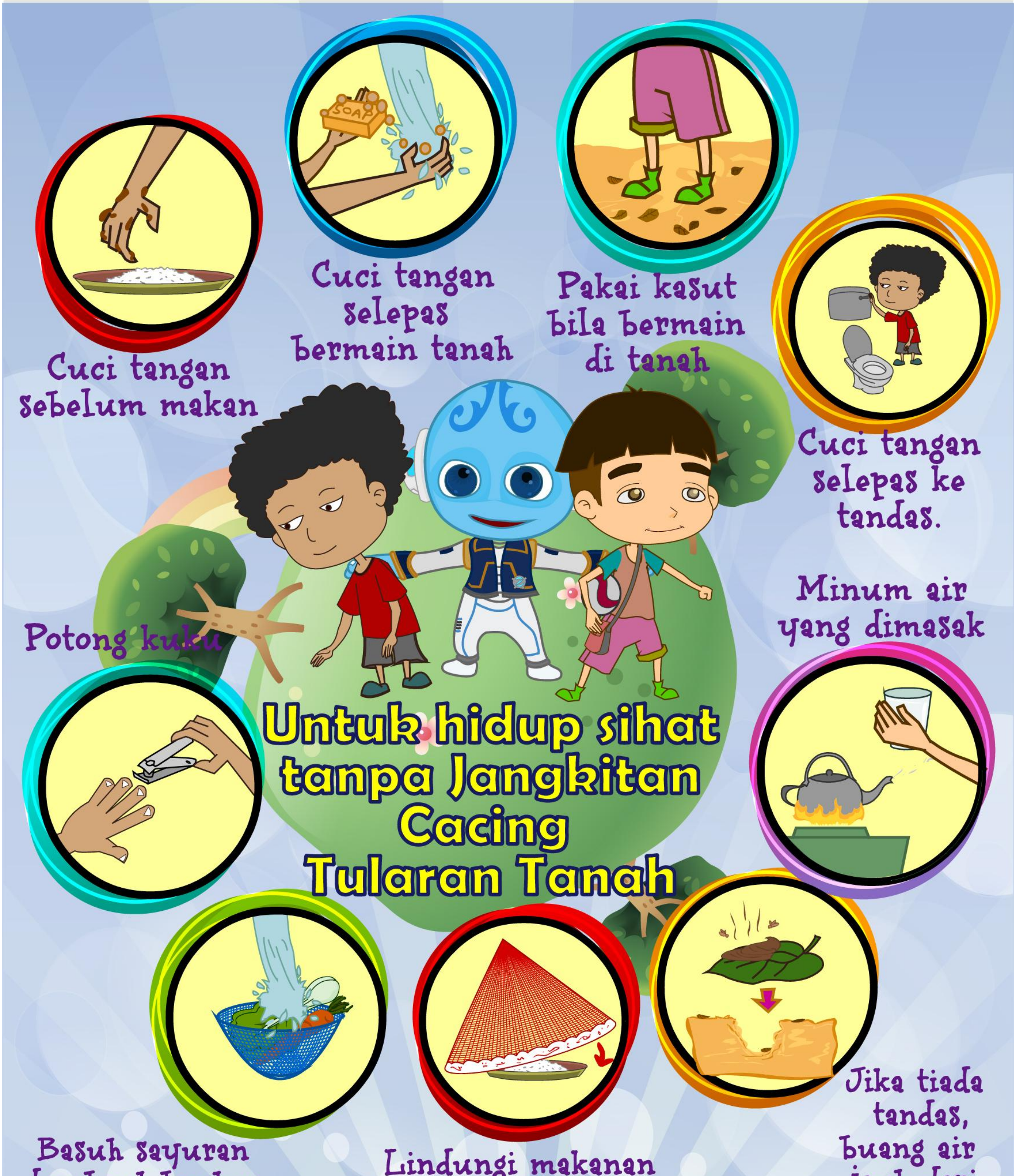

Health Education Learning Package to control Soil-Transmitted Helminthiasis among Orang Asli Schoolchildren

Project team: Assoc. Prof. Dr. Hesham M. Al-Mekhlafi
Professor Dr. Rohela Mahmud
Assoc. Prof. Dr. Yvonne AI Lim
Dr. Ahmed K. Al-Delaimy

Department of Parasitology, Faculty of Medicine, University of Malaya

Contact: halmekhlafi@yahoo.com, halmekhlafi@um.edu.my

UNIVERSITI MALAYA
The Leader in Research & Innovation

In collaboration with:

JAKOA
JABATAN KEMAJUAN ORANG ASLI

animagis sdn bhd
