# Supplementary material for: Developing and evaluating health education learning package (HELP) to control soil-transmitted helminth infections among Orang Asli children in Malaysia
Source: Parasit Vectors. 2014 Sep 2;7:416. doi: 10.1186/1756-3305-7-416 (PMC4261692; doi:10.1186/1756-3305-7-416)
Supplement: Supplementary file 2 — Additional file 2: The comic book used by this study. (PDF 11 MB) [file 13071_2014_1598_MOESM2_ESM.pdf]

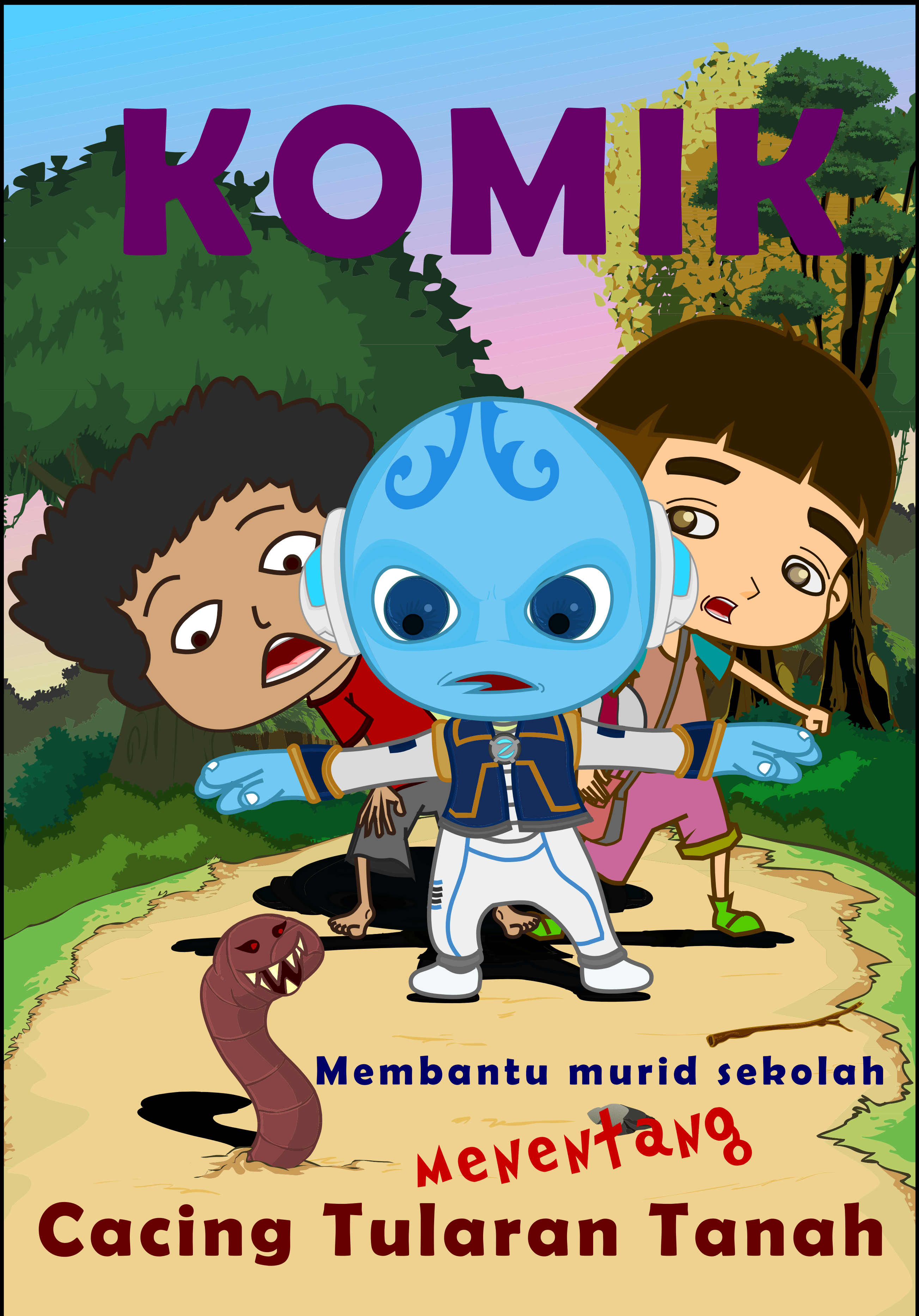
KOMIK
Membantu murid sekolah
menentang
Cacing Tularan Tanah

Basuh Tangan Dengan Betul

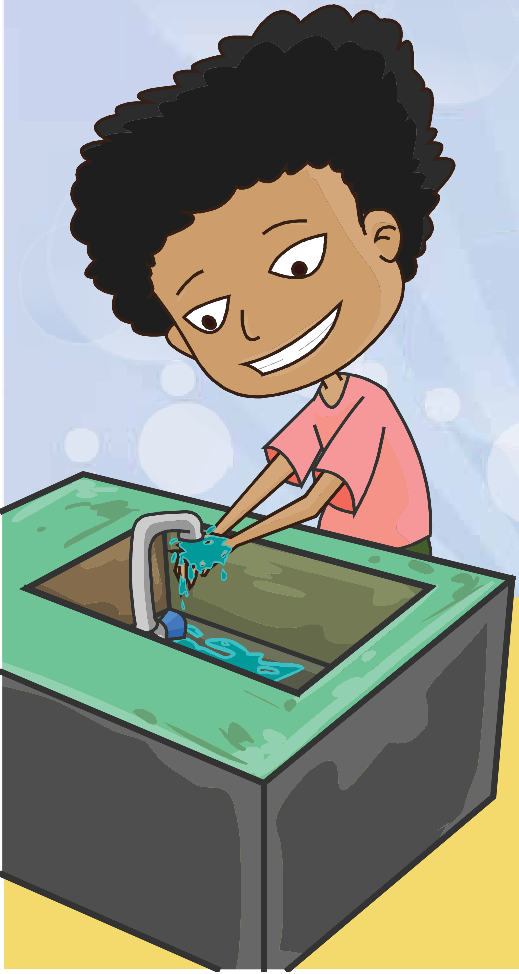

Mari kawan-kawan kita amalkan.
Guna cara betul untuk basuh tangan.
Ada tujuh langkah perlu kita amal.
Agar jauh dari penyakit dan kuman.

Pertama, guna sabun dengan secukupnya,
barulah harum wangi tangan kita.

Kedua, gosokkan sabun di tapak tangan,
biar sabun kena di semua bahagian.

Ketiga, gosok di setiap jari-jari kita,
jangan lupa di celah kuku-kuku juga.

Keempat, gosok kuku di tapak tangan,
supaya tanggal semua kotoran.

Kelima, gosok sabun di belakang tangan
agar tangan bersih keseluruhan

Keenam, basuh dengan air secukupnya
hindari semua kotoran dan bakteria

Terakhir, keringkan tangan guna kain bersih
barulah selesai semuanya

Jagalah kebersihan tangan kita,
sebab tangan guna untuk banyak perkara.
Guna untuk pegang, untuk angkat,
untuk makan.
Bila tangan bersih baru orang suka kita.

Lagu dan Lirik oleh : Abu Zaharen

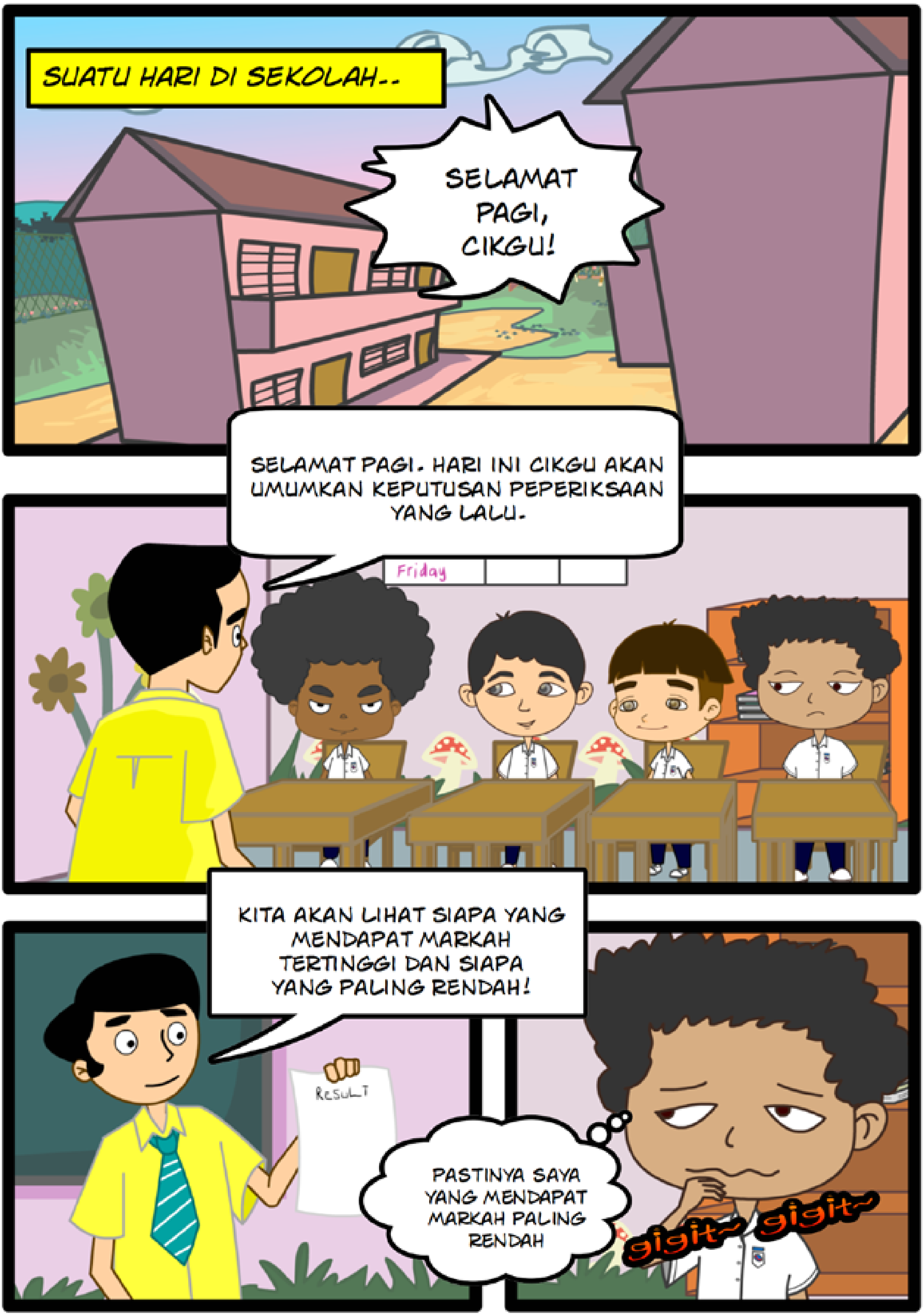
SUATU HARI DI SEKOLAH..
SELAMAT PAGI, CIKGU!
SELAMAT PAGI. HARI INI CIKGU AKAN UMUMKAN KEPUTUSAN PEPERIKSAAN YANG LALU.
Friday
KITA AKAN LIHAT SIAPA YANG MENDAPAT MARKAH TERTINGGI DAN SIAPA YANG PALING RENDAH!
RESULT
PASTINYA SAYA YANG MENDAPAT MARKAH PALING RENDAH
gigit~ gigit~

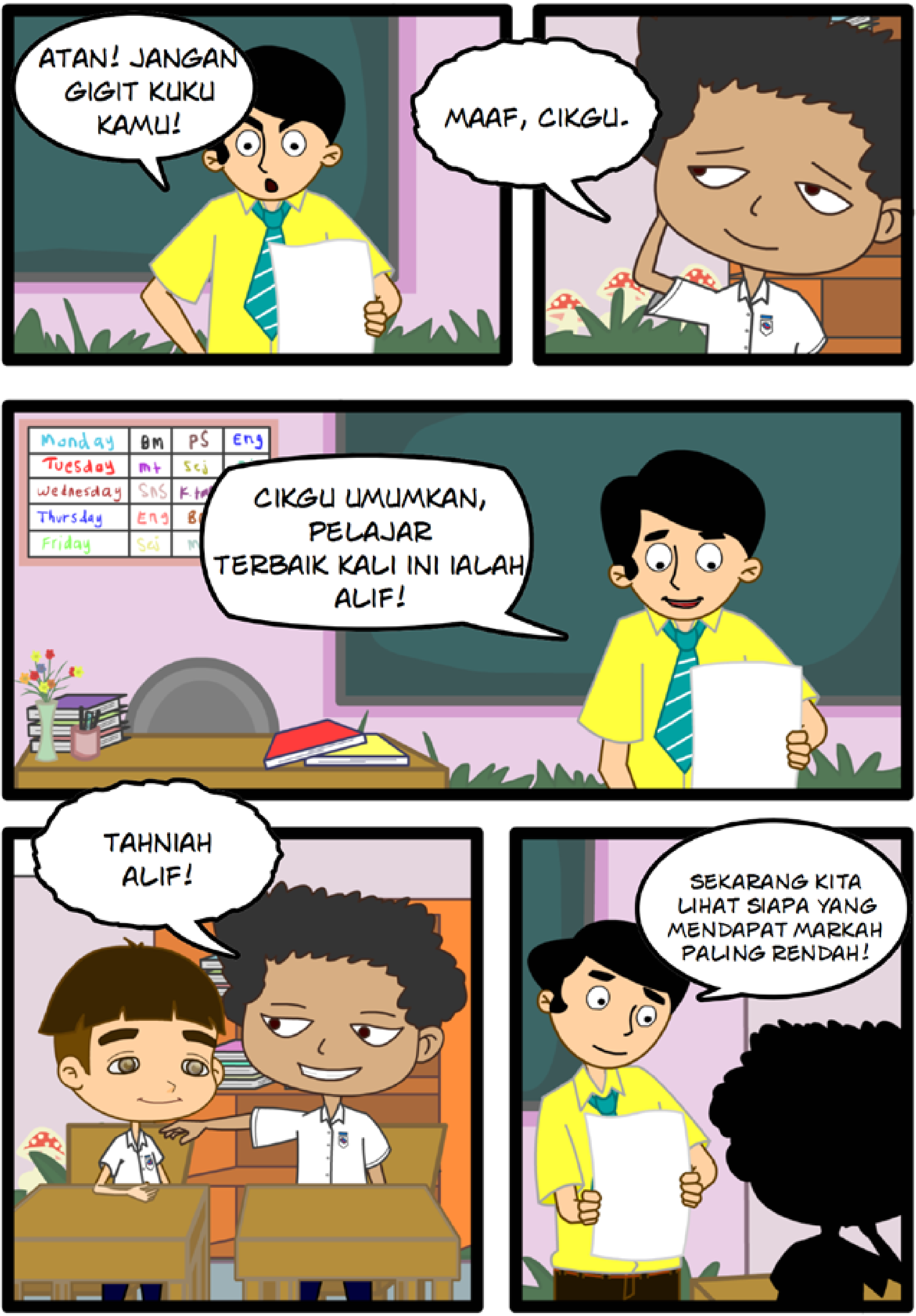
ATAN! JANGAN GIGIT KUKU KAMU!
MAAF, CIKGU.
Monday BM PS Eng
Tuesday Sci
Wednesday
Thursday Eng
Friday
CIKGU UMUMKAN, PELAJAR TERBAIK KALI INI IALAH ALIF!
TAHNIAH ALIF!
SEKARANG KITA LIHAT SIAPA YANG MENDAPAT MARKAH PALING RENDAH!

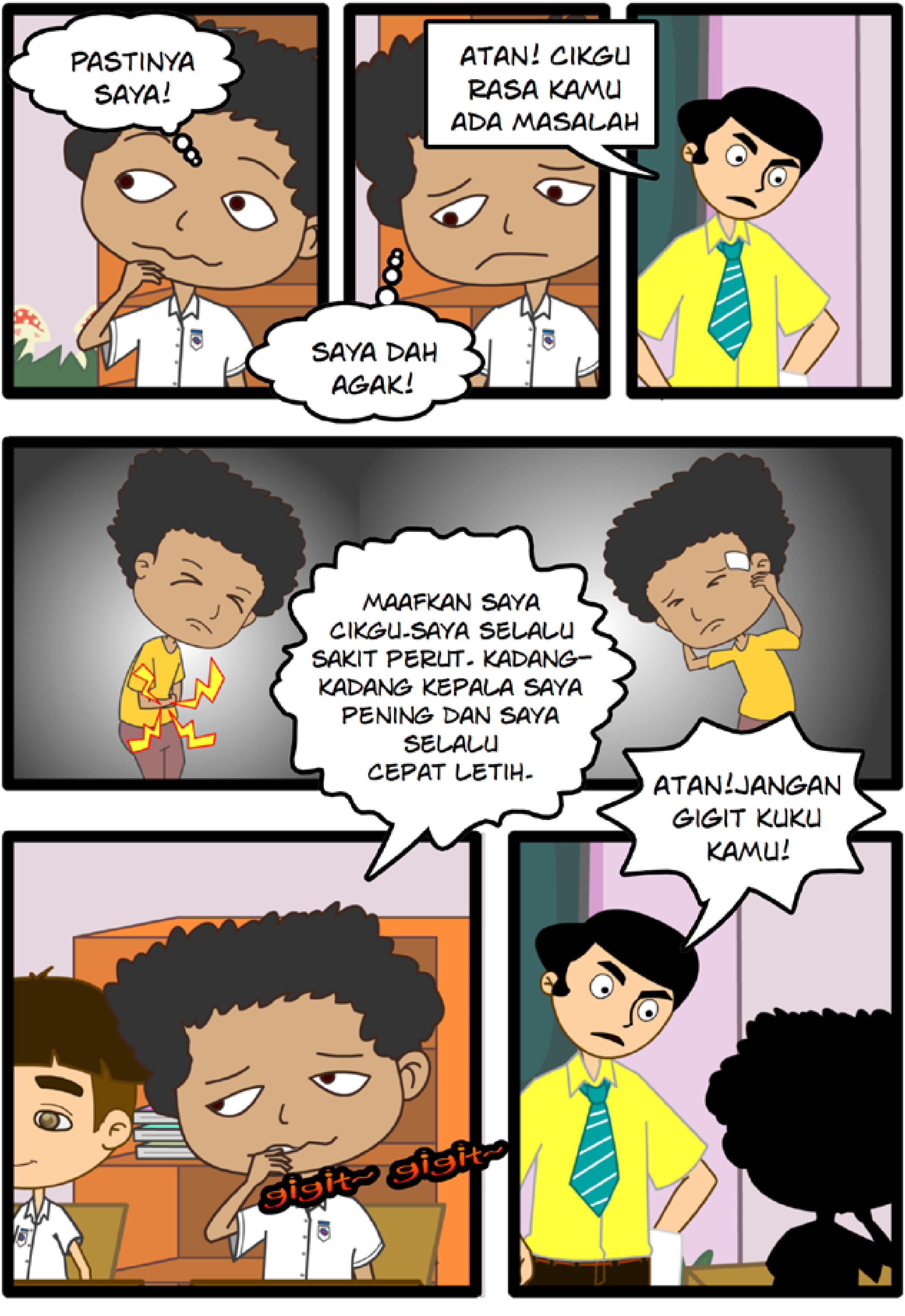
PASTINYA SAYA!
ATAN! CIKGU RASA KAMU ADA MASALAH
SAYA DAH AGAK!
MAAFKAN SAYA CIKGU. SAYA SELALU SAKIT PERUT. KADANG-KADANG KEPALA SAYA PENING DAN SAYA SELALU CEPAT LETIH.
gigit~ gigit~
ATAN! JANGAN GIGIT KUKU KAMU!

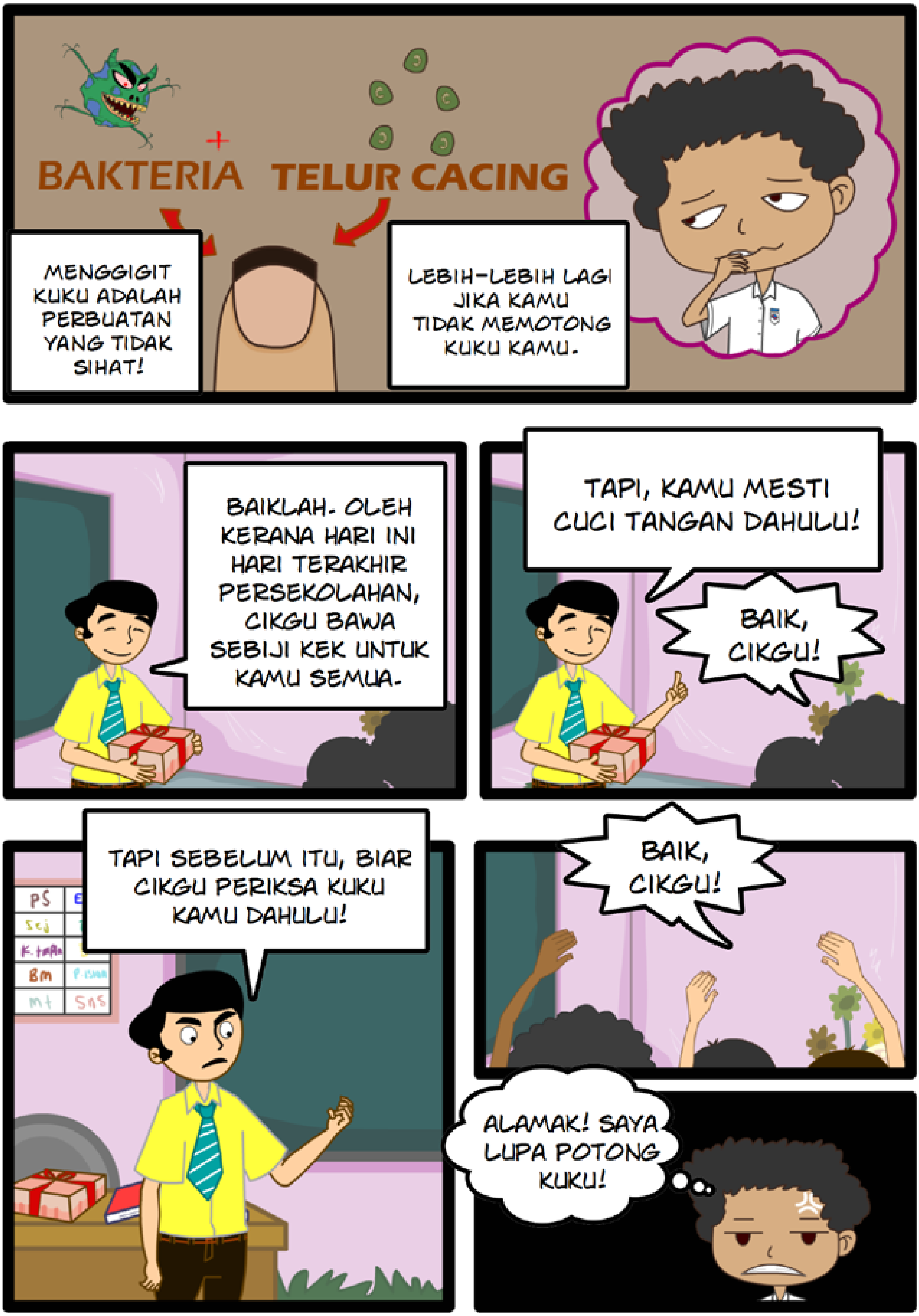
BAKTERIA
+
TELUR CACING
MENGGIGIT KUKU ADALAH PERBUATAN YANG TIDAK SIHAT!
LEBIH-LEBIH LAGI JIKA KAMU TIDAK MEMOTONG KUKU KAMU.
BAIKLAH. OLEH KERANA HARI INI HARI TERAKHIR PERSEKOLAHAN, CIKGU BAWA SEBIJI KEK UNTUK KAMU SEMUA.
TAPI, KAMU MESTI CUCI TANGAN DAHULU!
BAIK, CIKGU!
TAPI SEBELUM ITU, BIAR CIKGU PERIKSA KUKU KAMU DAHULU!
BAIK, CIKGU!
ALAMAK! SAYA LUPA POTONG KUKU!

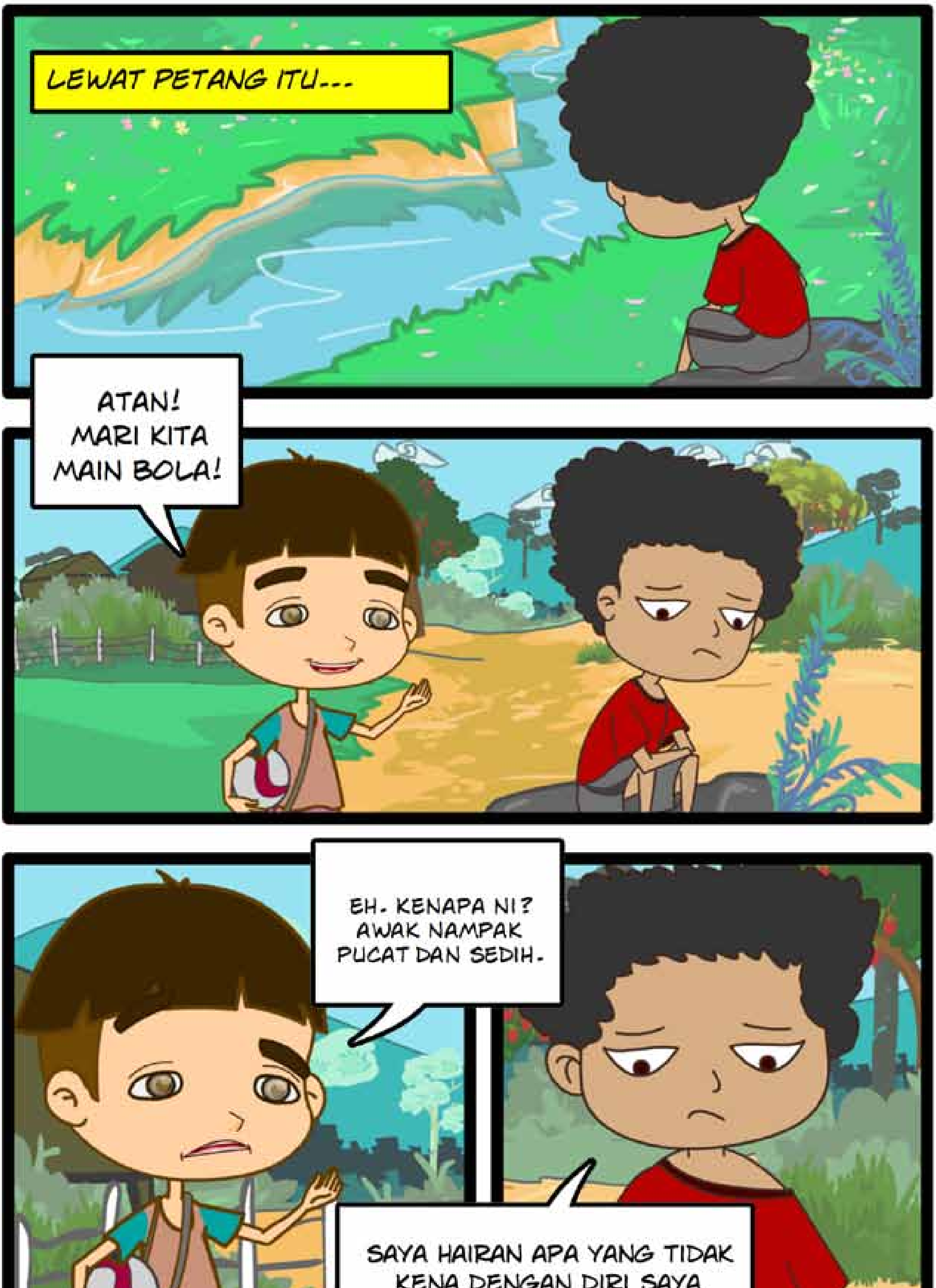
LEWAT PETANG ITU...
ATAN! MARI KITA MAIN BOLA!
EH. KENAPA NI? AWAK NAMPAK PUCAT DAN SEDIH.
SAYA HAIRAN APA YANG TIDAK KENA DENGAN DIRI SAYA

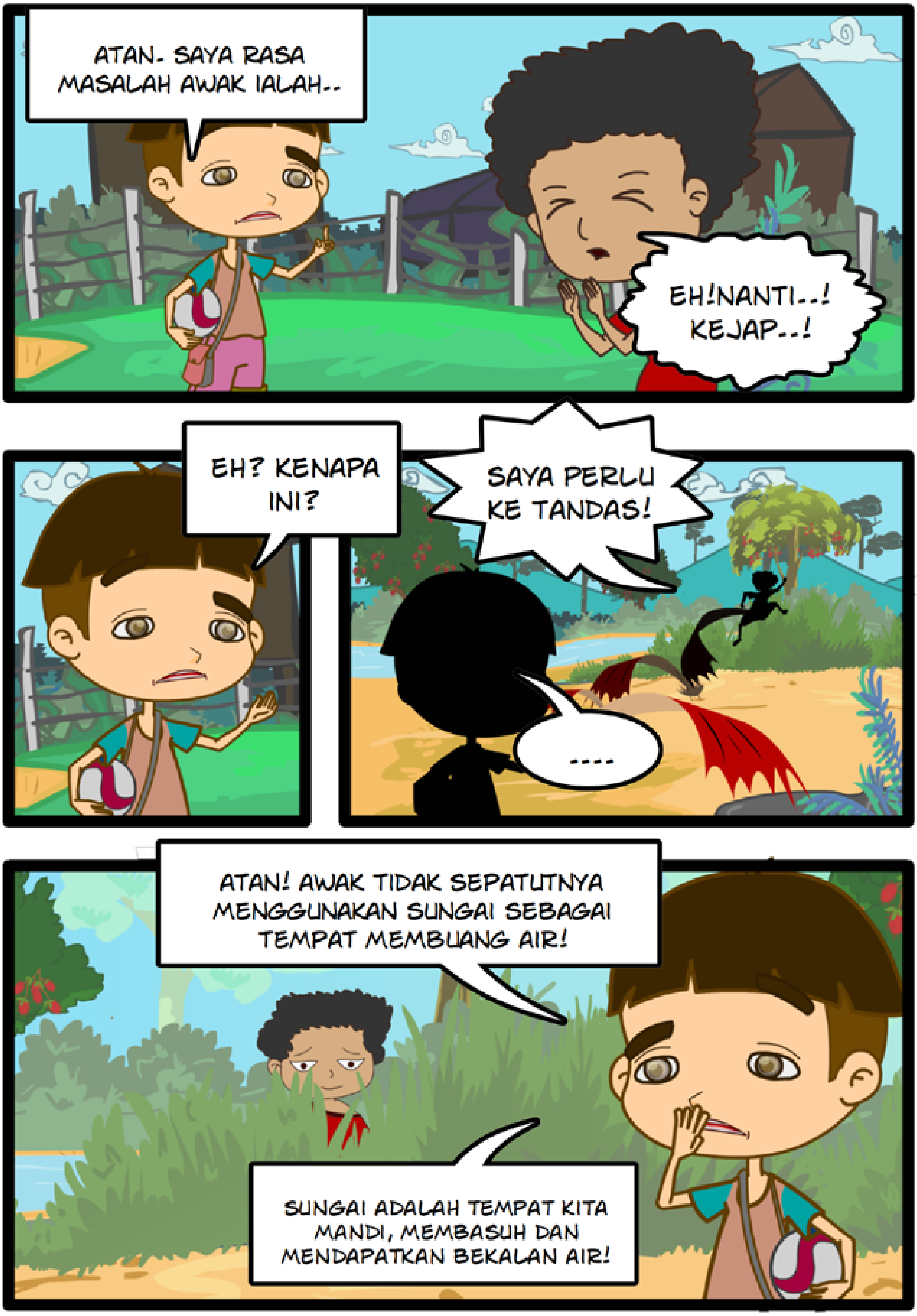
ATAN. SAYA RASA MASALAH AWAK IALAH..
EH!NANTI..! KEJAP..!
EH? KENAPA INI?
SAYA PERLU KE TANDAS!
....
ATAN! AWAK TIDAK SEPATUTNYA MENGGUNAKAN SUNGAI SEBAGAI TEMPAT MEMBUANG AIR!
SUNGAI ADALAH TEMPAT KITA MANDI, MEMBASUH DAN MENDAPATKAN BEKALAN AIR!

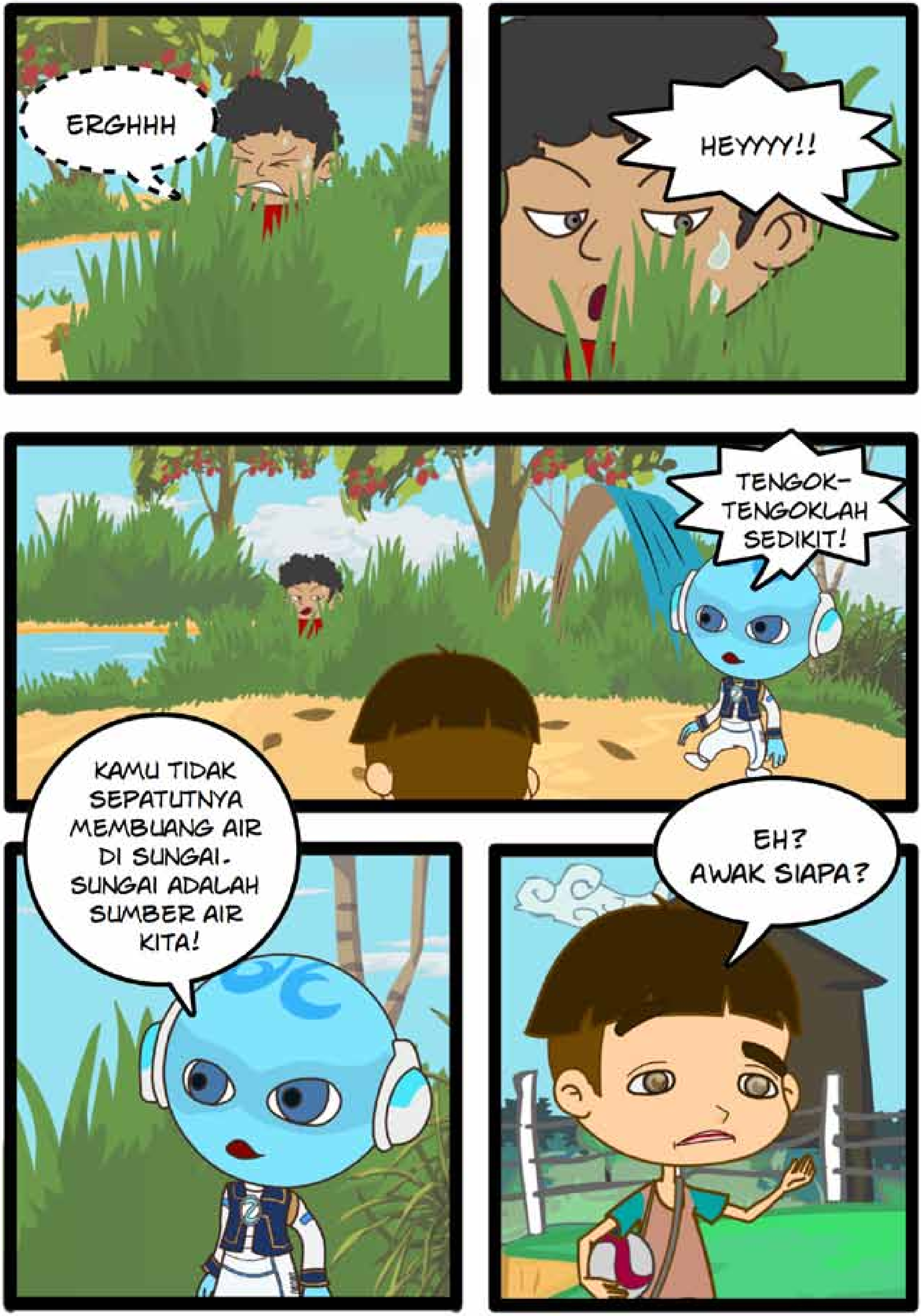
ERGHHH
HEYYYY!!
TENGOK-TENGOKLAH SEDIKIT!
KAMU TIDAK SEPATUTNYA MEMBUANG AIR DI SUNGAI. SUNGAI ADALAH SUMBER AIR KITA!
EH? AWAK SIAPA?

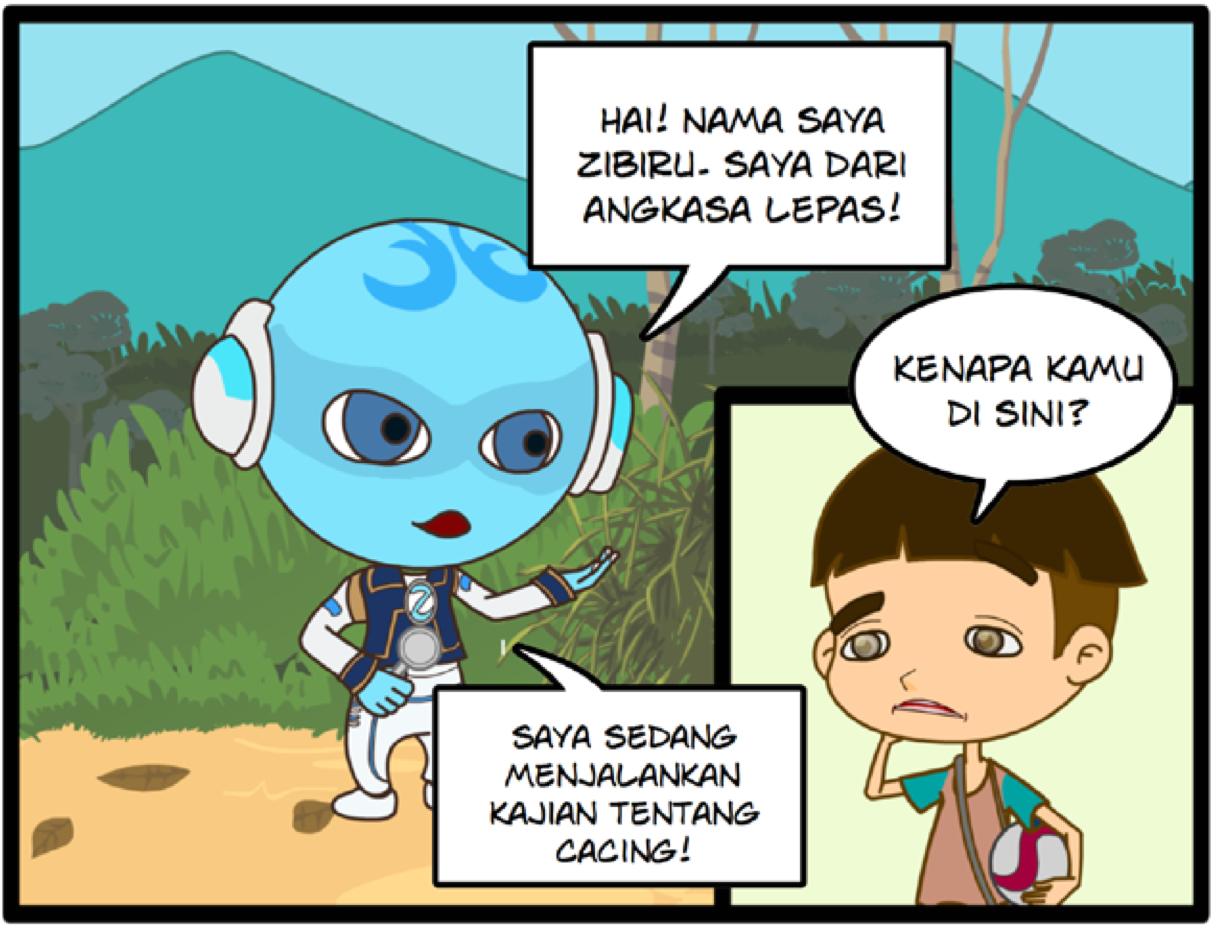
HAI! NAMA SAYA ZIBIRU. SAYA DARI ANGKASA LEPAS!
KENAPA KAMU DI SINI?
SAYA SEDANG MENJALANKAN KAJIAN TENTANG CACING!

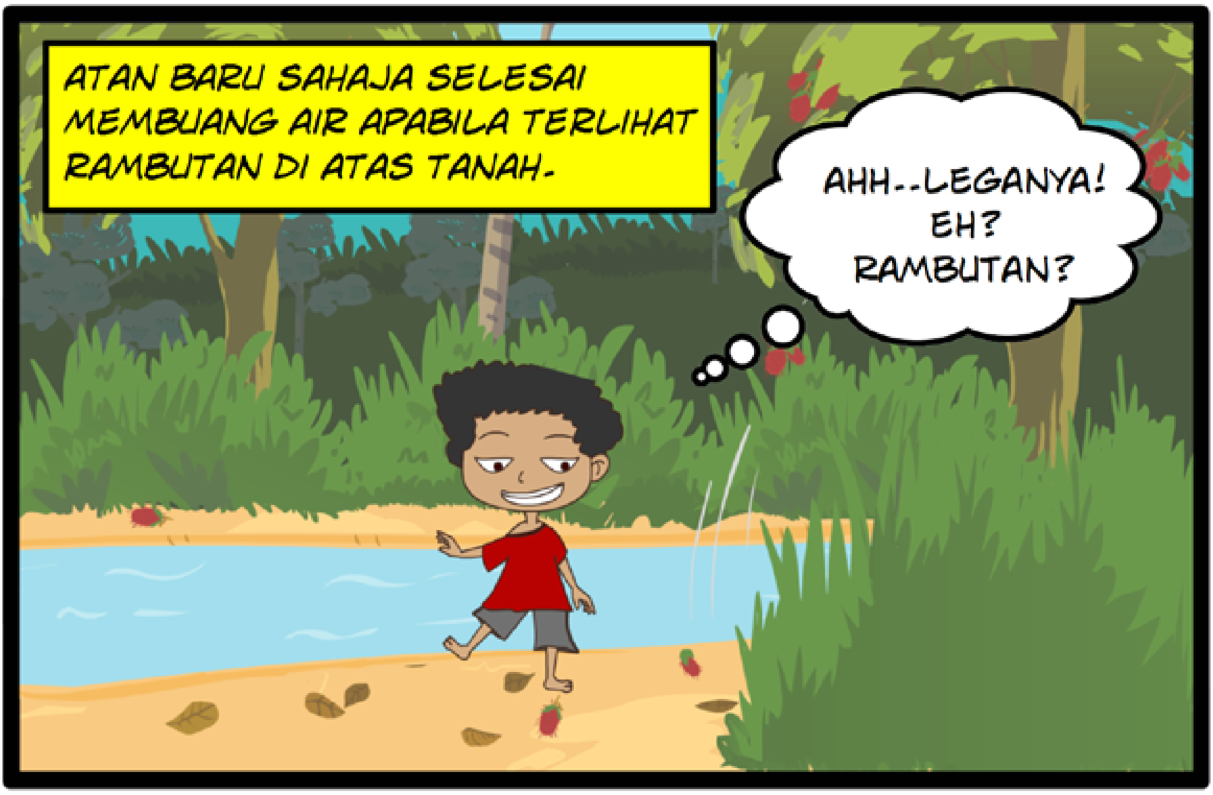
ATAN BARU SAHAJA SELESAI MEMBUANG AIR APABILA TERLIHAT RAMBUTAN DI ATAS TANAH.
AHH..LEGANYA! EH? RAMBUTAN?

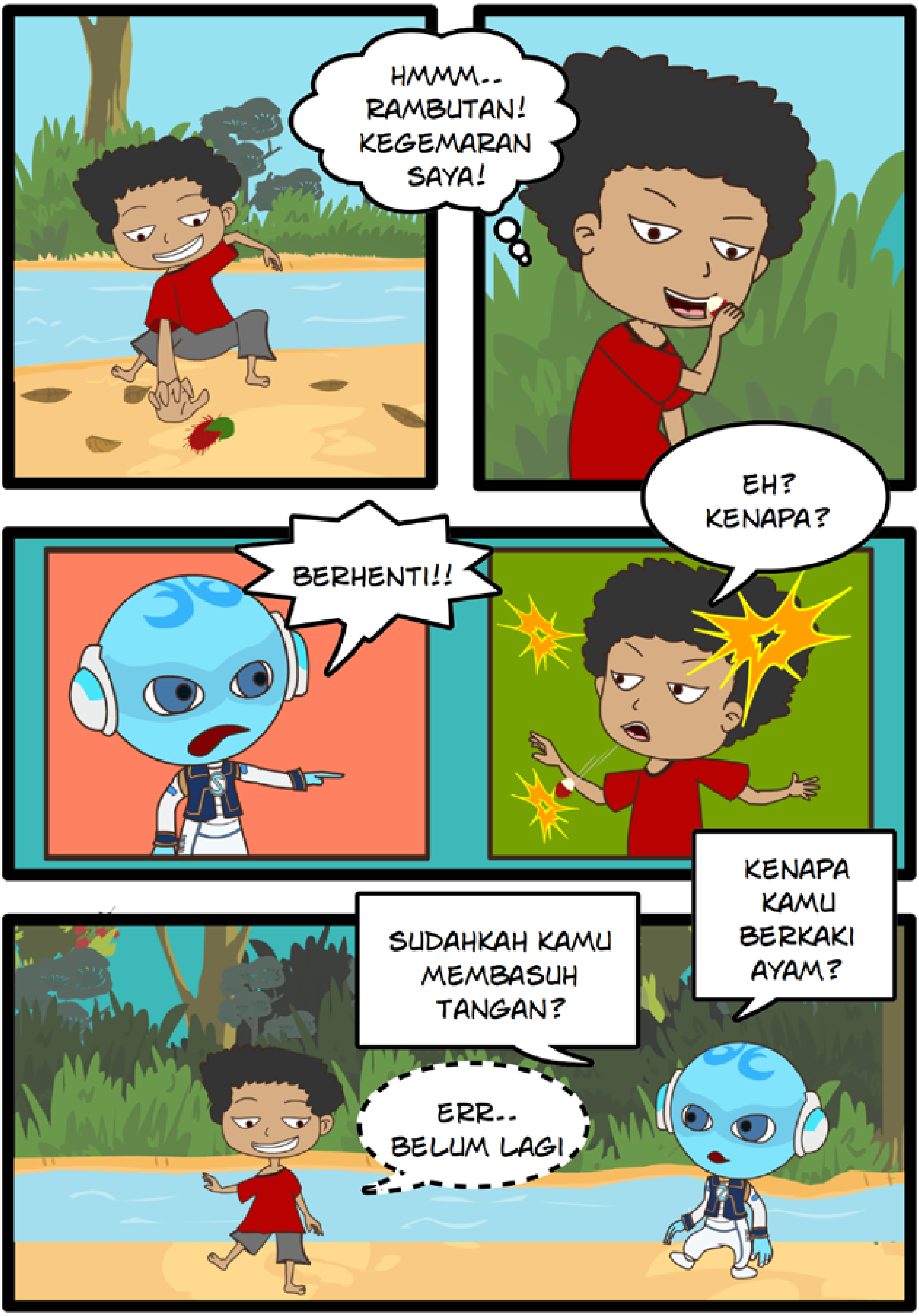
HMMM..
RAMBUTAN! KEGEMARAN SAYA!
BERHENTI!!
EH? KENAPA?
KENAPA KAMU BERKAKI AYAM?
SUDAHKAH KAMU MEMBASUH TANGAN?
ERR.. BELUM LAGI

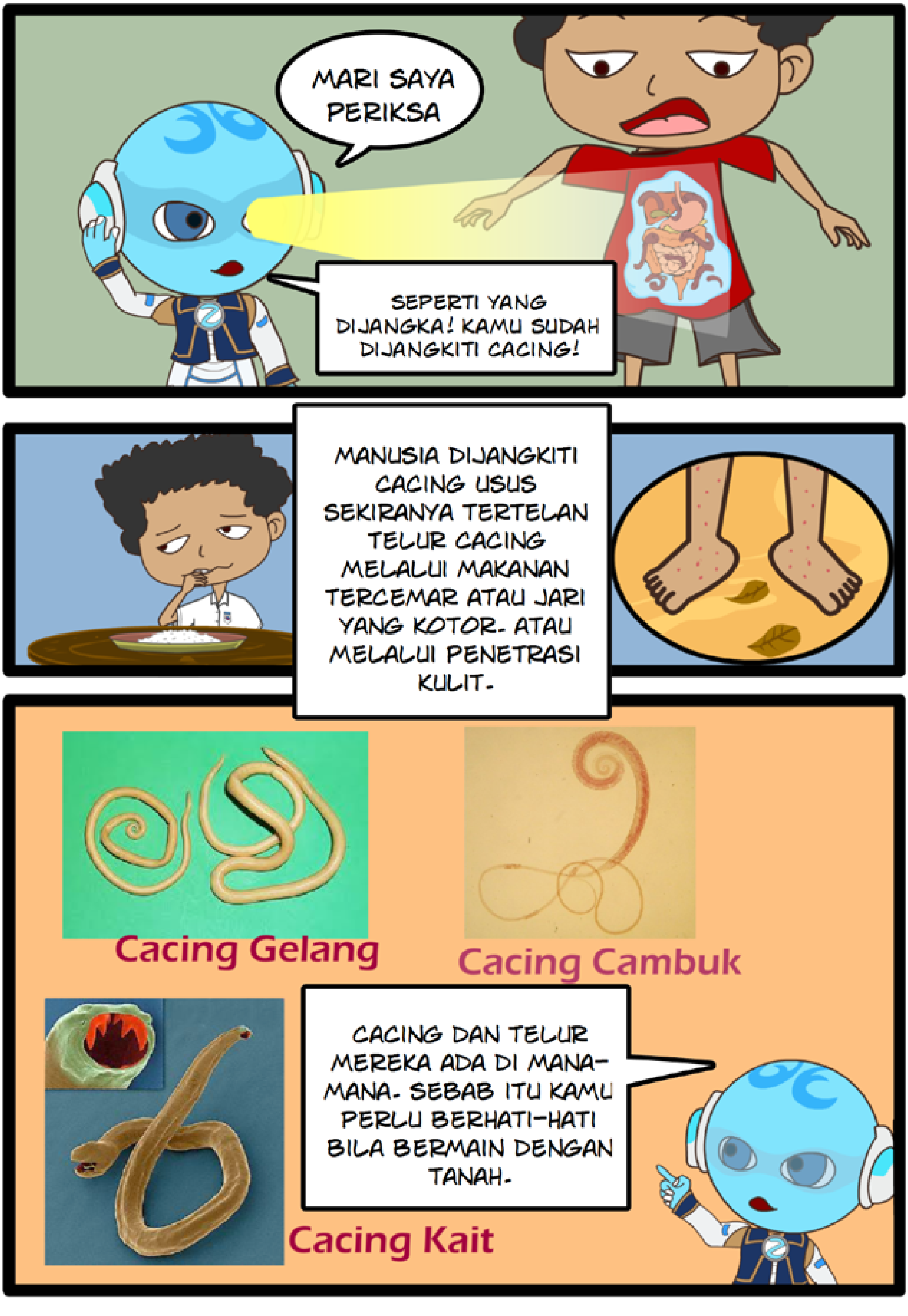
MARI SAYA PERIKSA
SEPERTI YANG DIJANGKA! KAMU SUDAH DIJANGKITI CACING!
MANUSIA DIJANGKITI CACING USUS SEKIRANYA TERTELAN TELUR CACING MELALUI MAKANAN TERCEMAR ATAU JARI YANG KOTOR. ATAU MELALUI PENETRASI KULIT.
Cacing Gelang
Cacing Cambuk
Cacing Kait
CACING DAN TELUR MEREKA ADA DI MANA-MANA. SEBAB ITU KAMU PERLU BERHATI-HATI BILA BERMAIN DENGAN TANAH.

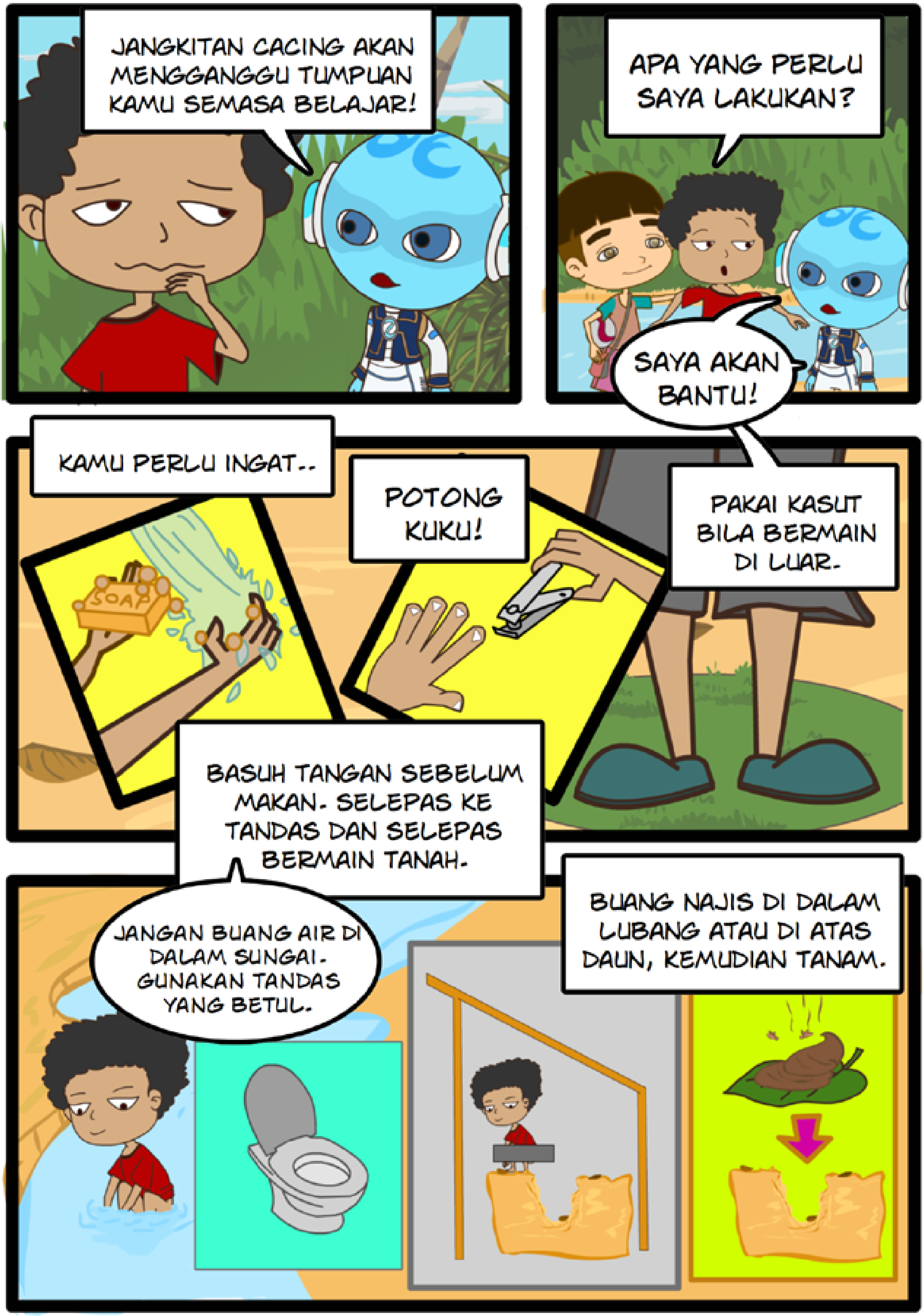
JANGKITAN CACING AKAN MENGGANGGU TUMPUAN KAMU SEMASA BELAJAR!
APA YANG PERLU SAYA LAKUKAN?
SAYA AKAN BANTU!
KAMU PERLU INGAT..
SOAP
POTONG KUKU!
PAKAI KASUT BILA BERMAIN DI LUAR.
BASUH TANGAN SEBELUM MAKAN. SELEPAS KE TANDAS DAN SELEPAS BERMAIN TANAH.
JANGAN BUANG AIR DI DALAM SUNGAI. GUNAKAN TANDAS YANG BETUL.
BUANG NAJIS DI DALAM LUBANG ATAU DI ATAS DAUN, KEMUDIAN TANAM.

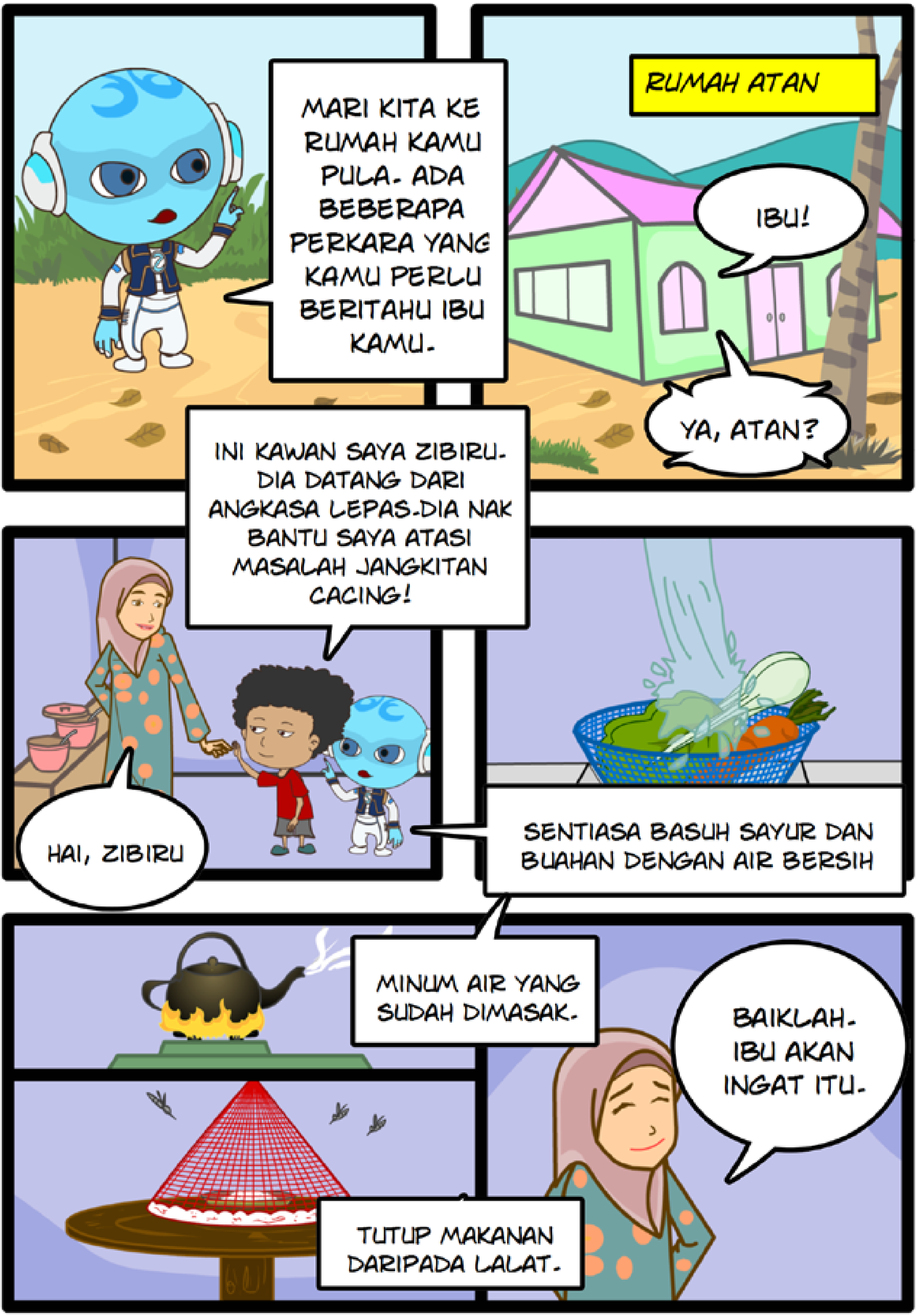
MARI KITA KE RUMAH KAMU PULA. ADA BEBERAPA PERKARA YANG KAMU PERLU BERITAHU IBU KAMU.
RUMAH ATAN
IBU!
YA, ATAN?
INI KAWAN SAYA ZIBIRU. DIA DATANG DARI ANGKASA LEPAS.DIA NAK BANTU SAYA ATASI MASALAH JANGKITAN CACING!
HAI, ZIBIRU
SENTIASA BASUH SAYUR DAN BUAHAN DENGAN AIR BERSIH
MINUM AIR YANG SUDAH DIMASAK.
TUTUP MAKANAN DARIPADA LALAT.
BAIKLAH. IBU AKAN INGAT ITU.

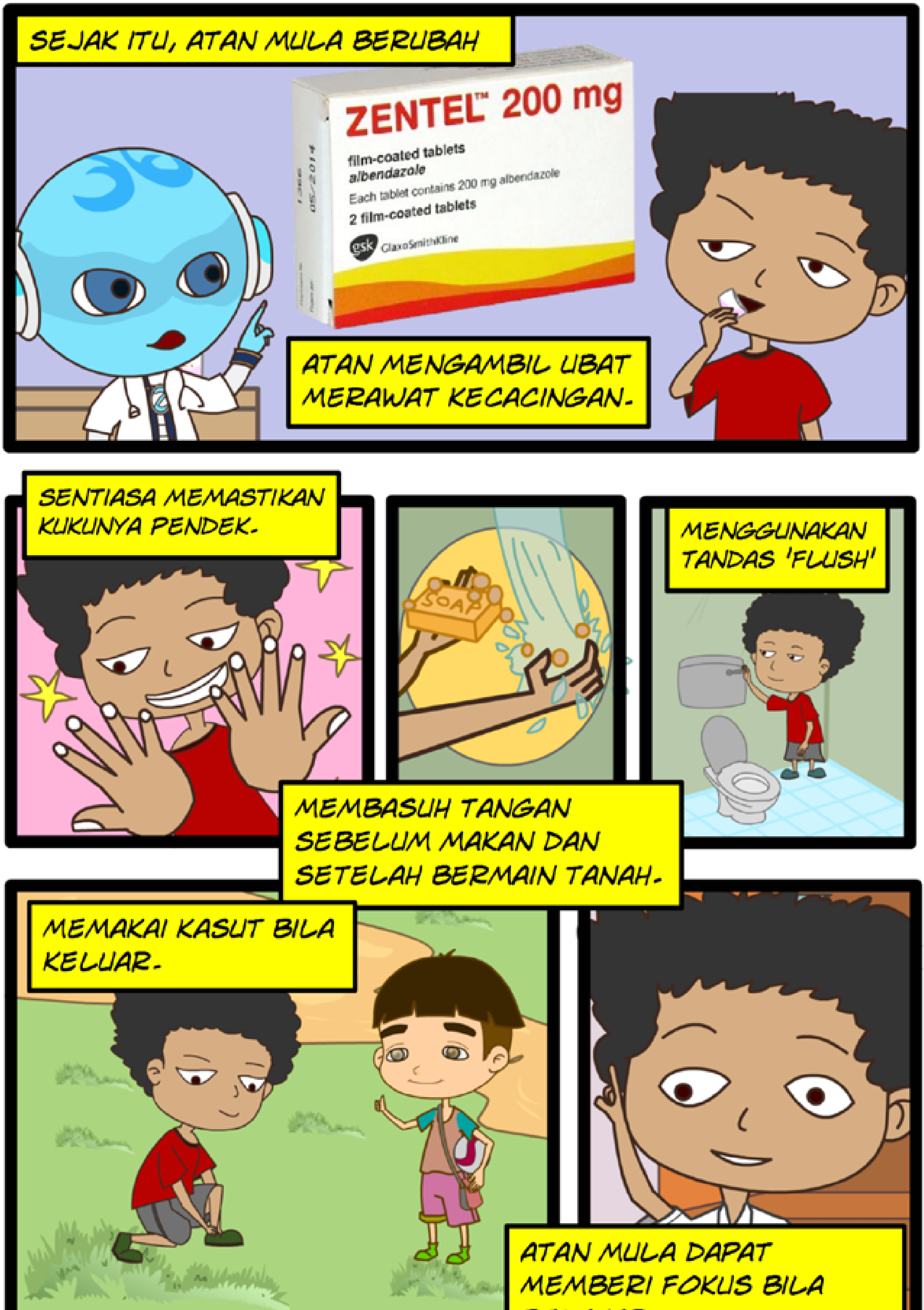
SEJAK ITU, ATAN MULA BERUBAH
ZENTEL™ 200 mg
film-coated tablets
albendazole
Each tablet contains 200 mg albendazole
2 film-coated tablets
gsk GlaxoSmithKline
ATAN MENGAMBIL UBAT MERAWAT KECACINGAN.
SENTIASA MEMASTIKAN KUKUNYA PENDEK.
SOAP
MENGGUNAKAN TANDAS 'FLUSH'
MEMBASUH TANGAN SEBELUM MAKAN DAN SETELAH BERMAIN TANAH.
MEMAKAI KASUT BILA KELUAR.
ATAN MULA DAPAT MEMBERI FOKUS BILA BELAJAR.

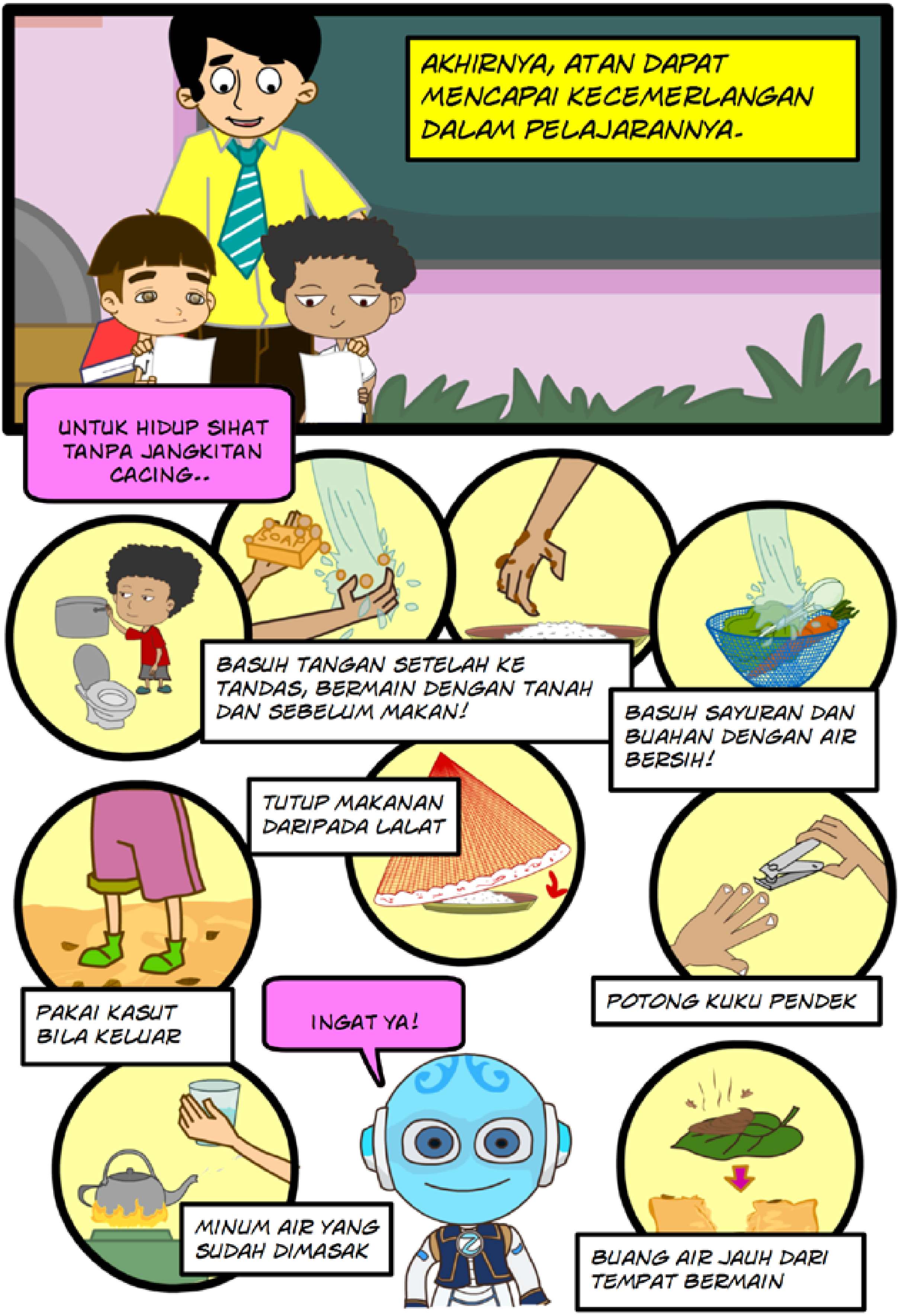
AKHIRNYA, ATAN DAPAT MENCAPAI KECEMERLANGAN DALAM PELAJARANNYA.
UNTUK HIDUP SIHAT TANPA JANGKITAN CACING..
SOAP
BASUH TANGAN SETELAH KE TANDAS, BERMAIN DENGAN TANAH DAN SEBELUM MAKAN!
BASUH SAYURAN DAN BUAHAN DENGAN AIR BERSIH!
TUTUP MAKANAN DARIPADA LALAT
PAKAI KASUT BILA KELUAR
POTONG KUKU PENDEK
INGAT YA!
MINUM AIR YANG SUDAH DIMASAK
BUANG AIR JAUH DARI TEMPAT BERMAIN

Tandakan (✓) pada amalan yang betul dan (X) pada amalan yang salah.

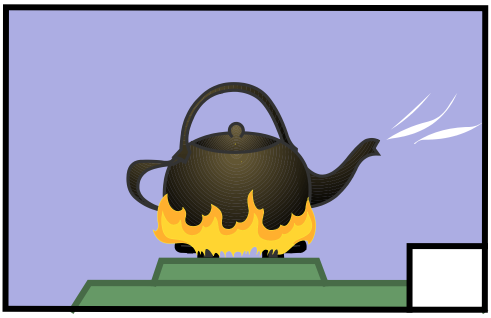

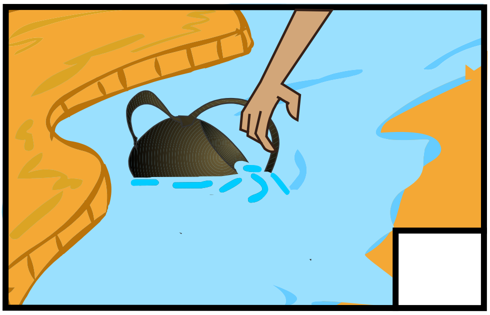

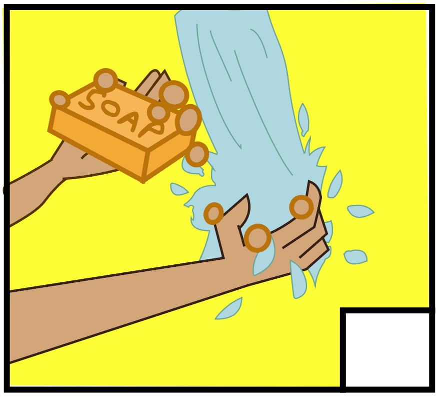

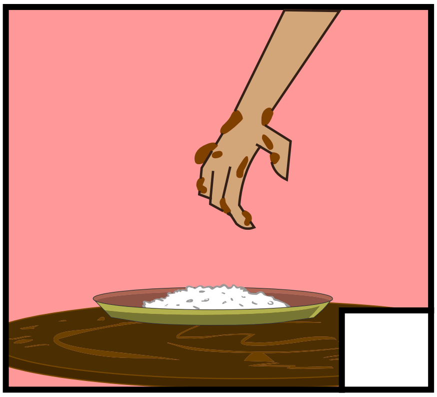

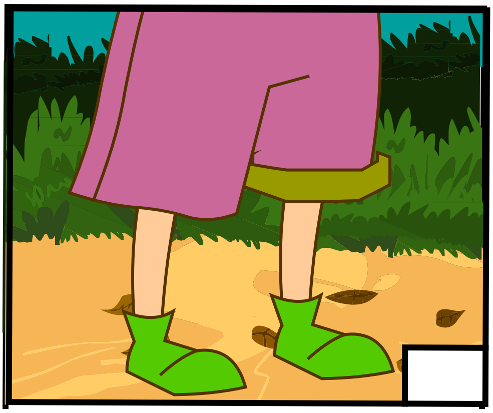

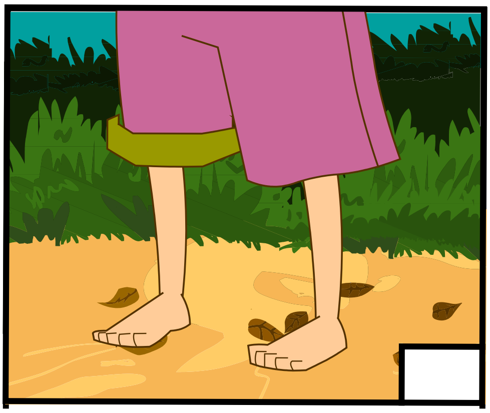

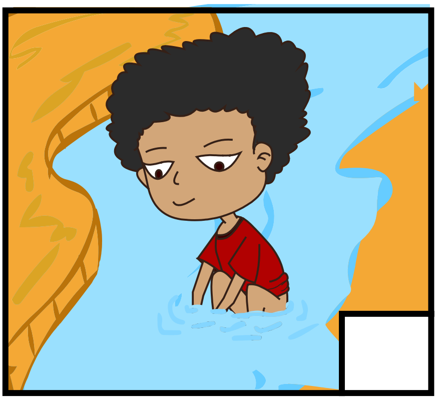

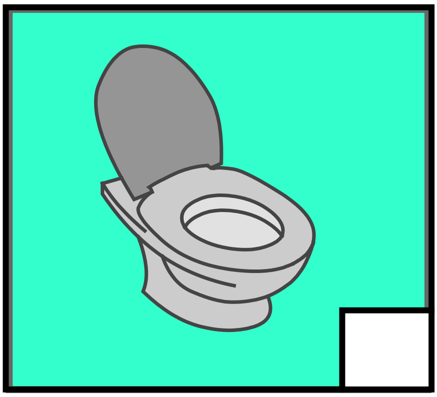

Warnakan Poster di bawah mengikut warna kesukaan adik-adik.

UNTUK MENGELAKKAN JANGKITAN

CACING TULARAN TANAH

PAKAILAH KASUT

BILA BERMAIN DI LUAR.

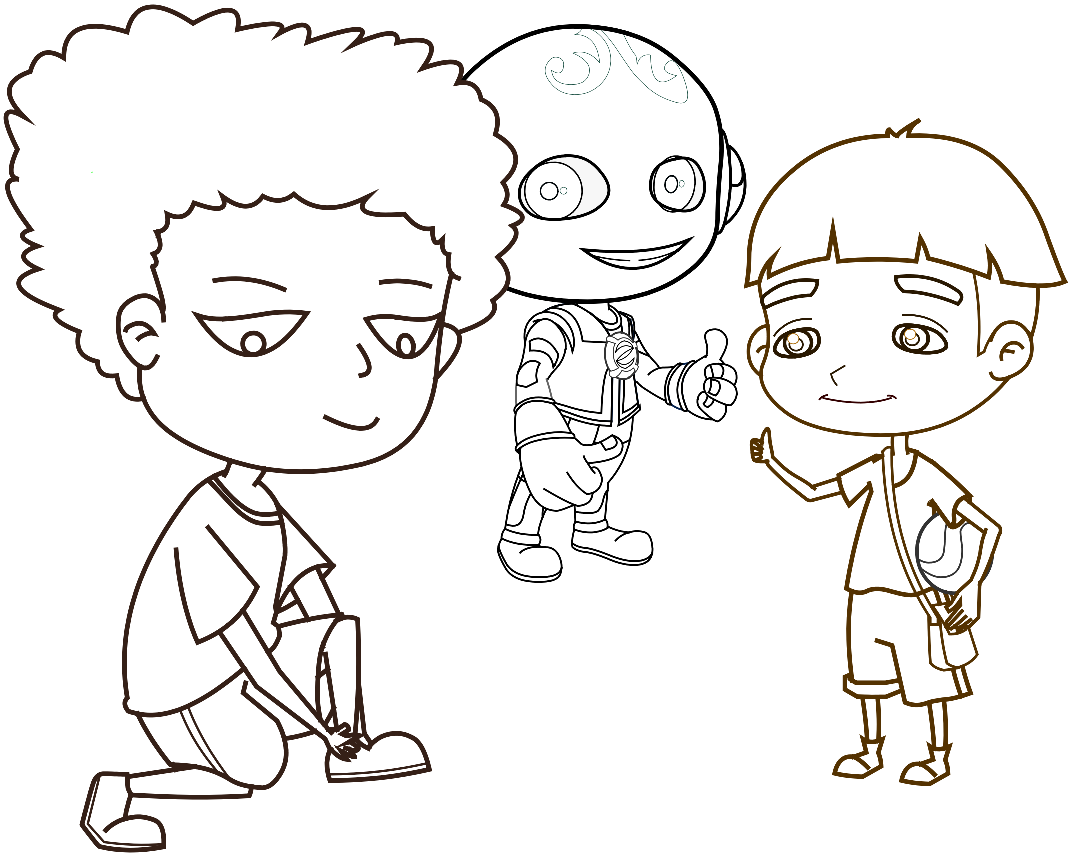

Jagalah Kebersihan Diri

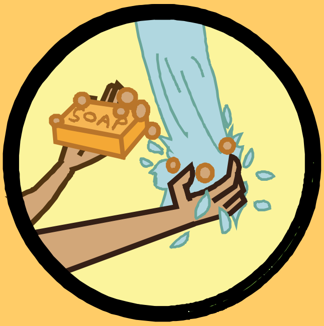

Marilah kita semua
jaga kebersihan diri kita
badan sihat orang pun suka
baru hidup kita ceria~
bangun pagi gosok gigi
mandi dgn air yang bersih
guna sabun cucikan badan
guna tuala bersih badan dikeringkan

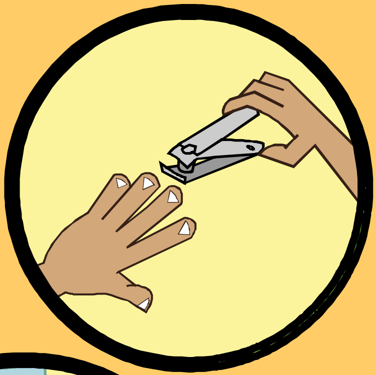

shampu rambut ketika mandi
selepas itu sikat rambut dengan rapi
pakai pakaian yang bersih
pasti orang rasa kasih
potong kuku sebelum panjang
untuk hindari bakteria

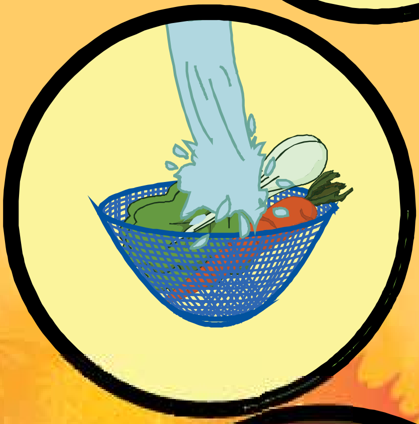

cuci tangan sebelum makan
supaya tidak dijangkiti kuman
makanlah buah dan sayuran
dan cucikan sebelum makan
supaya kita sihat dan kuat
dan tidak dijangkiti penyakit

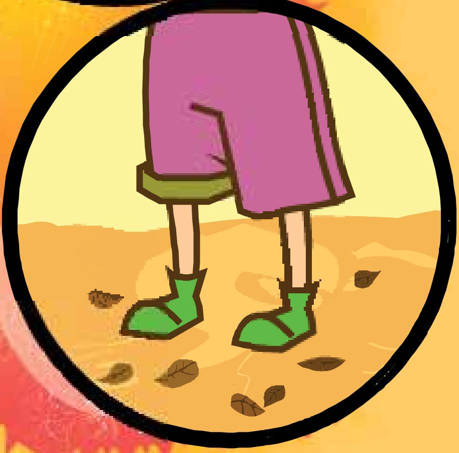

pakai kasut yang bersih
bila hendak keluar rumah
untuk melindung kaki kita
dari cacing dan kotoran

(solo)
Amalkan cara hidup sihat
menjaga tubuh dan penampilan
baru hidup penuh berkat
dan penuh senyuman
dan penuh senyuman

lagu dan lirik : Abu Zaharen

Health Education Learning Package to control Soil-Transmitted Helminthiasis among Orang Asli Schoolchildren

UNIVERSITI MALAYA

The Leader in Research & Innovation

Project team: Assoc. Prof. Dr. Hesham M. Al-Mekhlafi
Professor Dr. Rohela Mahmud
Assoc. Prof. Dr. Yvonne Al Lim
Dr. Ahmed K. Al-Delaimy

Department of Parasitology, Faculty of Medicine,
University of Malaya

Contact: halmekhlafi@yahoo.com,
halmekhlafi@um.edu.my

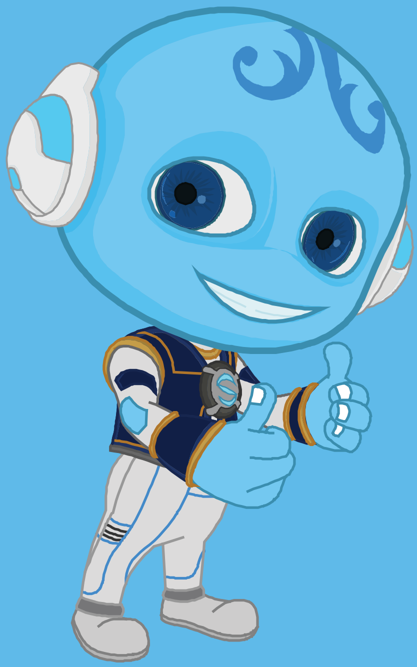

In collaboration with:

JAKOA
JABATAN KEMAJUAN ORANG ASLI

animägis sdn bhd

©2013 HAKCIPTA TERPELIHARA
